# Autism Spectrum Disorder and Gender Dysphoria/Incongruence. A systematic Literature Review and Meta-Analysis

**DOI:** 10.1007/s10803-022-05517-y

**Published:** 2022-05-20

**Authors:** Aimilia Kallitsounaki, David M. Williams

**Affiliations:** School of Psychology, University of Kent, Keynes College, CT2 7NP Canterbury, Kent, United Kingdom

**Keywords:** Autism spectrum disorder, Gender dysphoria, Gender identity, Meta-analysis, Literature review

## Abstract

The suggested overlap between autism spectrum disorder (ASD) and gender dysphoria/incongruence (GD/GI) has been much disputed. This review showed a relationship between ASD traits and GD feelings in the general population and a high prevalence of GD/GI in ASD. Our meta-analyses revealed that the pooled estimate of the prevalence of ASD diagnoses in GD/GI people was 11% (*p* < .001) and the overall effect size of the difference in ASD traits between GD/GI and control people was significant (*g* = 0.67, *p* < .001). Heterogeneity was high in both meta-analyses. We demonstrated that the chances that there is not a link between ASD and GD/GI are negligible, yet the size of it needs further investigation.

**Supplementary Information:**

The online version contains supplementary material available at 10.1007/s10803-022-05517-y.

Autism spectrum disorder (ASD) is a neurodevelopmental condition diagnosed based on significant challenges with social-communication and a restricted, repetitive pattern of interests and behavior (American Psychiatric Association, 2013). It affects approximately 1% of the worldwide population (e.g., Lai et al., 2014) and is associated with high rates of co-occurring conditions (e.g., depression and anxiety; Joshi et al., 2013; Lever & Geurts, 2016; Vohra et al., 2017) as well as with a higher risk of suicide than the general population (Cassidy et al., 2014; Hirvikoski et al., 2016). In recent years, several clinicians and researchers have postulated that ASD overlaps with gender dysphoria (GD) or gender incongruence (GI; e.g., Strang, Janssen, et al., 2018; van der Miesen, Cohen-Kettenis, et al.,^2018^).

GD, formally known as gender identity disorder (GID; American Psychiatric Association, 2000), is a psychiatric condition characterized by an incongruence between one’s birth-assigned sex and one’s experienced/reported gender, which is accompanied by clinically significant distress about this incongruence (American Psychiatric Association, 2013). GI is a more general term that describes a condition in which a person’s experienced/reported gender does not align with their birth-assigned sex (Butler, 2020). We should note here that not all GI individuals experience GD (Olson-Kennedy et al., 2016). As to the prevalence of co-occurring mental health conditions, research has shown that it is significantly higher in GD/GI adults than in the general population (e.g., Dhejne et al., 2016; Zucker et al., 2016). Research has also shown that GD/GI children and adults are at increased risk of self-harm and suicidality (e.g., Aitken et al., 2016; Cerel et al., 2021; de Graaf et al., 2020). Given the well-established increased risk of mental health conditions and suicidal behavior both in ASD and GD/GI, it could be argued that the co-occurrence of these conditions imposes a particularly distressful burden on an individual (George & Stokes, 2018a; Hall et al., 2020). Therefore, it is important to study and understand this link.

The number of publications on the suggested overlap between ASD and GD/GI has more than doubled in the last two years, reflecting the increased attention this topic has received from clinicians, researchers, as well as the lay press (e.g., Seaman, 2016; Strang, Janssen, et al., 2018). Yet, it remains debated whether evidence supports this hypothesis (Fortunato et al., 2021; Turban & van Schalkwyk, 2018; Turban, 2018). Several reviews of the literature on the co-occurrence of ASD and GD/GI have been conducted, but most of these were based on a very small number of studies published prior to 2016 (Glidden et al., 2016; van der Miesen et al., 2016; van Schalkwyk et al., 2015; Wood & Halder, 2014).

More recently, Øien et al. (2018) identified and catalogued studies on the co-occurrence of ASD and GD, but did not attempt to answer any research questions. In contrast, Thrower et al. (2020) conducted a systematic review of the literature to investigate whether there is an overrepresentation of ASD diagnoses/caseness (and attention deficit hyperactivity disorder) in people with GD. They found that the prevalence of ASD diagnoses in this population was increased (range: 6–26%), but this was not examined statistically. This leaves a critical gap in the literature, which we propose to fill in the current article.

To examine whether the evidence indicates a link a between ASD and GD/GI, we adopted two approaches in the current article. In Part 1 we appraised the existing literature concerning the co-occurrence of ASD and GD/GI, including not only studies conducted among autistic and GD/GI individuals but also studies that examined the suggested overlap in the general population. It is important to note here that the features of ASD represent a single continuum that is known as “broad autism phenotype”. People with a diagnosis of ASD fall at the high end of this continuum and people with low levels of ASD traits fall at the low end of it (e.g., Bolton et al., 1994; Goldberg et al., 2005; Le Couteur et al., 1996; Murphy et al., 2000; Ronald et al., 2006). On this basis, it has been argued that important information about ASD can be obtained by investigating individual differences in ASD traits and their relationship to other phenomena among people from the general population (e.g., Lind et al., 2020). In Part 2 we conducted the first meta-analyses of studies of ASD diagnoses and ASD traits. Our aim was to report the first pooled prevalence estimate of ASD diagnoses in GD/GI people and examine the hypothesis that GD/GI people have elevated levels of ASD traits.^1^

## Part 1: ASD and GD/GI: A Systematic Literature Review

## Method

To perform the systematic literature review and meta-analyses (see Part 2), we followed the Preferred Reporting Items for Systematic Reviews and Meta-Analyses (PRISMA) checklist guidelines where possible (Moher et al., 2015). A systematic literature search was conducted on Web of Science, Pub Med, and PsycINFO by the first author of this article. Studies of interest were those examining the overlap between ASD/ASD traits and GD/GI. To identify articles published prior to October 2020, we used combinations of the following search terms: “autism, autism spectrum disorder, autistic traits, autistic, ASD, Asperger syndrome, GD, transgender, gender dysphoric, GID, transsexualism, transgenderism, sex reassignment, GI, non-binary/nonbinary, gender variance, gender non-conformity/gender nonconformity, and gender diversity”, using the Boolean AND operator.

**Fig. 1:**
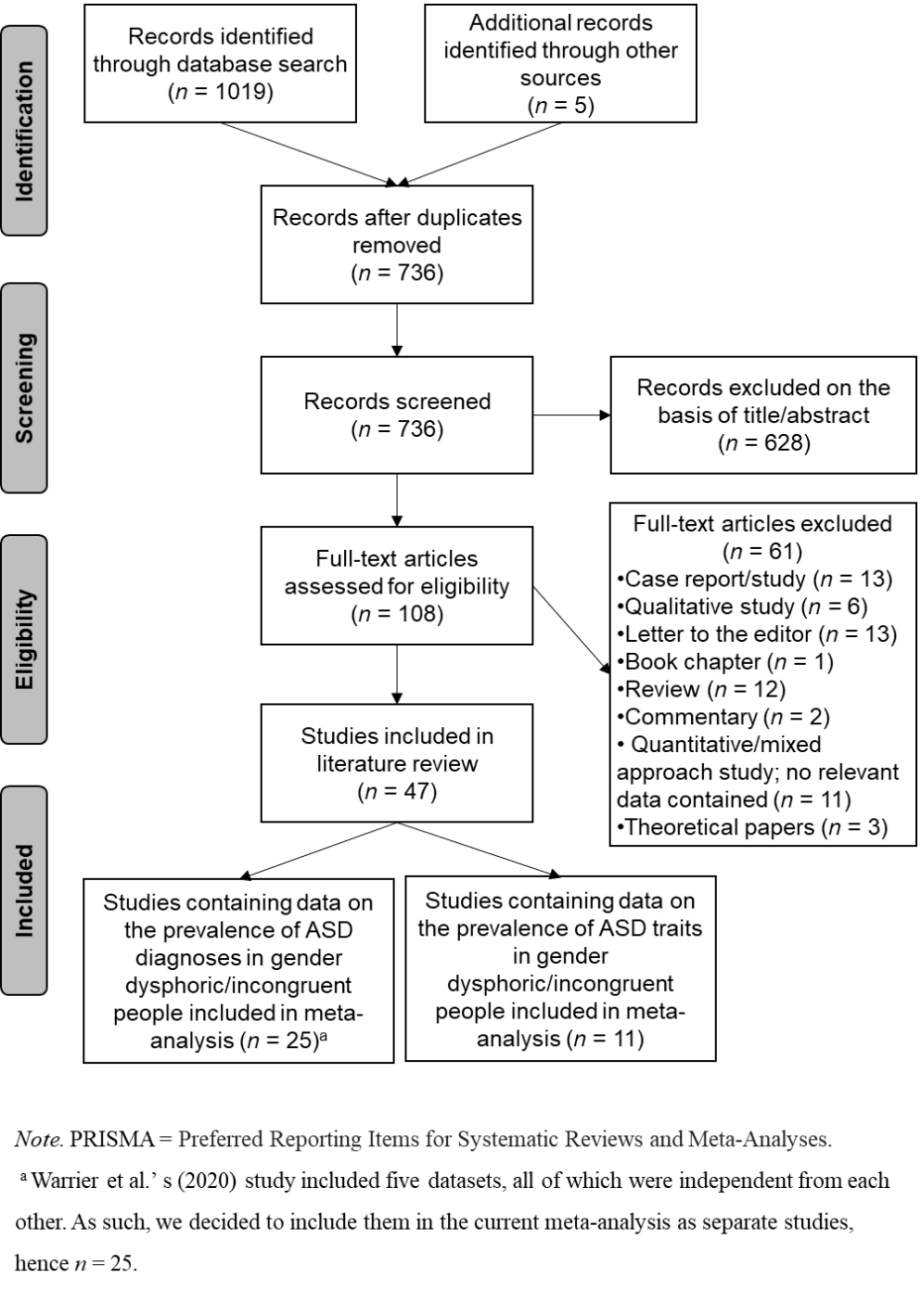
PRISMA Flow Chart Illustrating Study Selection Process

Articles that were considered eligible were those that examined either the relationship between ASD traits and GD/GI in the general population, the prevalence of GD/GI in autistic cohorts, or the prevalence of ASD diagnoses/caseness/traits in GD/GI cohorts. Please note that when articles reported evidence about the prevalence of ASD diagnoses, GD/GI participants had to have—self-report—a formal diagnosis of ASD (including Asperger’s syndrome, autism, and pervasive developmental disorder). Also, when articles reported evidence about the prevalence of ASD traits in GD/GI cohorts, evidence from a control group (either primary or secondary) should have also been reported. Lastly, to be considered eligible, articles should have reported quantitative results, been published in peer-reviewed journals, and been written in English. As Fig. 1 illustrates, the study selection was conducted in two stages.

In the first stage, we screened articles by title and abstract for relevance. At this stage, we excluded proceedings papers and grey literature articles, including dissertations. In the second stage, we evaluated the suitability for inclusion based on the full-text articles. Studies that reported nonquantitative results, such as reviews, were excluded at this stage (see Table S1). Forty-seven articles met all the eligibility criteria and were included in the current systematic literature review.

## Results

We were able to identify 1,019 studies through the database search and five studies through other sources. After duplicates were removed, we screened 736 studies and excluded irrelevant ones. From the remaining 108 full-text articles, we excluded 61 studies because they did not fulfil the criteria described in the method section. Hence, 47 studies that contained information on the overlap between ASD and GD/GI were included in the current systematic literature review. Of these studies, five were conducted with children, 13 with children and adolescents, two with adolescents, two with children, adolescents, and adults, nine with adolescents and adults, and 16 with adults (for a summary of the identified studies, see Table S2).

### Studies in the General Population

Shumer et al. (2015) were the first to report that elevated ASD traits in children *or* in mothers predicted high gender nonconformity in children. Nabbijohn et al. (2019) found a positive and significant association between ASD traits and gender variance in a sample of non-autistic children. The greater the number of ASD traits reported by parents in these children, the more parent-reported gender variance in these children. Interestingly, in a sample of non-autistic adults with no clinically significant levels of ASD traits, George and Stokes (2018b) reported a positive and significant association between the number of self-reported ASD traits and the number of concurrent GD feelings. Kallitsounaki and Williams (2020) successfully replicated this finding in cisgender people from the general population and they extended this finding further by reporting a significant association between ASD traits and *recalled* gender-typed behavior (i.e., behavior that is considered stereotypically characteristic of a specific gender and recalled from childhood). That is, the higher the number of self-reported ASD traits by an individual, the more they reported experiencing current GD feelings and the less they recalled childhood gender-typed behavior. Results were successfully replicated in an independent sample by Kallitsounaki et al. (2021), highlighting the existence of a robust and reliable link between ASD traits, on the one hand, and current GD feelings and recalled childhood gender-typed behavior, on the other hand.

### Studies in the Autistic Population

Janssen et al. (2016), May et al. (2017), and Strang et al. (2014) used the item 110 from the Child Behavior Checklist (CBCL) to investigate cross-gender wishes (i.e., wishes to be of opposite binary gender). They all found that parents of autistic children endorsed this item more frequently, compared to parents of nonreferred (control) children. Adopting a similar methodology, van der Miesen, Hurley, et al. (2018) examined cross-gender wishes in autistic adolescents and adults, using item 110 of the Youth Self-Report and the Adult Self-Report. This item also measures endorsement of the wish to be the binary gender opposite to birth-assigned sex. The study reported that autistic adolescents were 2.12 times more likely to endorse this item for themselves than were nonreferred adolescents. Likewise, autistic adults were 2.46 times more likely to endorse the item than nonreferred adults. Although this single item approach has been frequently used to investigate gender variance in autistic individuals, it is not entirely free of criticism. It can be argued that the item 110 taps ideation rather than behavior. Therefore, it cannot be considered equivalent to a gender evaluation in which behaviors, wishes, and roles are all assessed. Also, Turban and van Schalkwyk (2018) offered an alternative explanation for the increased prevalence of the wish to be the binary gender opposite to birth-assigned sex observed in autistic people. They suggested that the well-established cognitive inflexibility in ASD might trigger *ephemeral* desires among autistic people to be the binary gender opposite to their birth-assigned sex.

Hisle-Gorman et al. (2019) has published the only study on the prevalence of a formal diagnosis indicating GD in children with a primary diagnosis of ASD. Collecting information from medical records, they conducted a matched case-cohort study and found that autistic children were over 4 times more likely to have a co-occurring diagnosis indicating GD than were non-autistic children. Furthermore, Nabbijohn et al. (2019) found that parents of autistic children reported significantly more gender variance in their children, compared to parents of non-autistic children. George and Stokes (2018b) utilized a standardized self-report measure of GD feelings to compare autistic adults with non-autistic adults (i.e., Gender Identity/Gender Dysphoria Questionnaire for Adolescents and Adults [GIDYQ-AA]; Deogracias et al., 2007). They found that the autistic group reported significantly more GD than the control group. However, it is important to note that the percentage of cisgender participants was 89.59% in the control group and 70.22% in the autistic group. Given the difference between the two groups, it could be argued that the inclusion of noncisgender people in the analysis could have artificially inflated the score of the autism group, creating a significant difference in GD feelings between autistic and control people. Replication of these findings await before strong conclusions can be drawn.

Research has also shown that autistic people, on average, report a more diverse range of gender identities than non-autistic individuals (Bejerot, & Eriksson, 2014; George & Stokes, 2018b). In keeping with these findings, Cooper et al. (2018) found that autistic individuals were significantly more likely to be GI and to have or plan to have a gender transition than non-autistic people. Indeed, Walsh et al. (2018) reported that 15% of autistic individuals who participated in their study reported trans and nonbinary identities. Surprisingly, also Dewinter et al. (2017) found that 15.4% of the autistic participants who participated in their study reported trans, nonbinary, and other/unknown gender identities. However, the latter study did not conduct a comparison with a population-based control group, so meaningful conclusions are difficult to be drawn from the results. Lastly, Pecora et al. (2020) found that autistic females were less likely to identify with their birth-assigned sex than non-autistic females.

### Studies in the GD/GI Population

#### Prevalence of ASD Diagnoses

To investigate the prevalence of ASD *diagnoses* in GD/GI cohorts, researchers have relied on (a) diagnostic instruments for ASD, (b) information obtained from patient files, and (c) self-reported ASD diagnosis. De Vries et al. (2010) published the first quantitative study on the prevalence of ASD diagnoses in GD individuals. To date, this is the only study that has employed a clinical diagnostic tool to identify clinically diagnosable ASD in a sample of GD/GI people. Specifically, de Vries et al. (2010) utilized the Dutch version of the Diagnostic Interview for Social and Communication Disorders-10th revision (DISCO-10) in 26 children and adolescents with suspected ASD who had been referred to a gender identity clinic for GD. The investigators reported that the incidence of ASD was 7.8% in the total sample of gender-referred individuals (*N* = 204).

Interestingly, among adolescents diagnosed with GID, 6.5% received a co-occurring diagnosis of ASD, whereas 1.9% of children with GID were diagnosed with ASD. Turban and van Schalkwyk (2018) argued that since ASD is a neurodevelopmental disorder that is usually detected early in development, the high rates of clinically diagnosable ASD found in adolescents and not children with GID indicate that the diagnostic tool de Vries at al. (2010) used did not tap “true” ASD characteristics. Instead, psychosocial issues, such as anxiety and depression, that are particularly common in adolescents with GD/GI might have artificially inflated adolescents’ scores on DISCO-10. However, it is important to stress here that in de Vries at al.’s (2010) study only 12.7% of the sample received a diagnostic assessment for ASD. As such, it remains unclear whether all autistic children and adolescents were detected.

Compared to diagnostic instruments for ASD, the analysis of prerecorded, patient-centered data has been more frequently used for the investigation of the prevalence of ASD diagnoses in GD/GI individuals. In chart reviews, the incidence of a diagnosis of ASD ranged from 3 to 21.3% in GD/GI children and adolescents (Becerra-Culqui et al., 2018; Chen et al., 2016; Chiniara et al., 2018; Holt et al., 2016; Khatchadourian et al., 2014; Leef et al., 2019; Nahata et al., 2017; Peterson et al., 2017; Shumer et al., 2016; Skagerberg et al., 2015; Spack et al., 2012) and from 4.8 to 7.8% in GD/GI adults (Cheung et al., 2018; Fielding & Bass, 2018; Heylens et al., 2018). When researchers relied upon self-reports, the percentage of gender-referred children and adolescents who reported possession of a diagnosis of ASD was 9.62% (Mahfouda et al., 2019), and the percentage of GI adults who reported possession of a diagnosis of ASD ranged from 2.7 to 82% (Jones et al., 2012; Kristensen & Broome, 2015; Murphy et al., 2020; Stagg & Vincent, 2019; Warrier et al., 2020).

#### Prevalence of ASD Caseness

To examine the prevalence of ASD *caseness* in GD/GI cohorts, researchers have relied on cutoff scores from ASD screening questionnaires. Studies have shown that the positive rates for ASD range from 14.5 to 68% in GD/GI children and adolescents (Akgül et al., 2018; Leef et al., 2019; Mahfouda et al., 2019; Shumer et al., 2016; Skagerberg et al., 2015; VanderLaan, Leef, et al., 2015; van der Miesen, de Vries, et al., 2018) and from 1.2 to 40.3% in GD/GI adults (Heylens et al., 2018; Jones et al., 2012; Kristensen & Broome, 2015; Lehmann et al., 2020; Murphy et al., 2020; Nobili et al., 2018; Nobili et al., 2020; Pasterski et al., 2014; Stagg & Vincent, 2019; Vermaat et al., 2018). However, the incidence of ASD caseness ranges widely depending on the cutoff scores used. For example, while Pasterski et al. (2014) found that 5.5% of transgender adults diagnosed with GD or GID scored ≥ 32 on the Autism-Spectrum Quotient (AQ-50; Baron-Cohen et al., 2001), suggesting clinically significant levels of ASD traits, Kristensen and Broome (2015) reported that 39% of gender-variant adults should be referred for an ASD diagnostic assessment as they scored > 6 on the AQ-10 (Allison et al., 2012).

### Prevalence of ASD Traits

To examine the prevalence of ASD *traits* in GD/GI cohorts, researchers have used parent-/self-report measures that index ASD characteristics. VanderLaan, Postema, et al. (2015) and Zucker et al. (2017) assessed circumscribed preoccupations and intense interests (one diagnostic feature of ASD) in children referred to a gender identity clinic, using the items 9 and 66 from the CBCL or Teacher’s Report Form. Both studies found elevated obsession in gender-referred children, compared to nonreferred and clinic-referred children. A significant increase in compulsion was reported in gender-referred children, compared to nonreferred children only.

Van der Miesen, de Vries, et al. (2018) used the Children’s Social Behavior Questionnaire to examine the prevalence of ASD traits in children diagnosed with GID. The study found significantly increased ASD traits in children with GID, compared to non-autistic control children. Another study compared children who satisfied the diagnostic criteria for GD to non-autistic controls, using the Social Responsiveness Scale (SRS; Akgül et al., 2018). The study found that the GD group had significantly more ASD traits than the control group. Notwithstanding, when Leef et al. (2019) used the same measure to compare children diagnosed with GID, GD, or gender identity disorder not otherwise specified (GID-NOS) with clinic-referred children no difference was found in parent-reported ASD traits. However, when they employed the Social Communication Questionnaire to tap ASD traits, they did find elevated ASD traits in children with GID, GD, or GID-NOS. While the results of the aforementioned studies seem to indicate an increased prevalence on ASD traits in GD children, the study of this topic in GD/GI adults is less clear.

In seven out of the eight identified studies that contained data on the prevalence of ASD traits in GD/GI adults, researchers have used the AQ to measure ASD traits. While Jones et al.’s (2012) study found that transgender men reported significantly more ASD traits than nonclinical males and females, no difference was found between transgender women and either nonclinical males or females. Nobili et al. (2018) and Murphy et al. (2020) replicated these findings. Likewise, Kung (2020) found that transgender men and nonbinary females reported significantly more ASD traits than control females from the general population. However, no difference was observed between either transgender women or nonbinary males and control males from the general population. Vermaat et al. (2018) found that birth-assigned females referred for GD reported significantly more ASD traits than control birth-assigned females. A significant difference in ASD traits was also found between birth-assigned females referred for GD and one of the three control samples of birth-assigned males they used (i.e., Dutch AQ scores), with birth-assigned females scoring higher than birth-assigned males. Birth-assigned males referred for GD scored significantly lower on the AQ than control birth-assigned males and no difference was found between birth-assigned males referred for GD and control birth-assigned females. It is also important to mention that in Pasterski et al.’s (2014) study, transgender men diagnosed with GD/GID scored higher on the AQ-50 than nonclinical birth-assigned females, but the difference was small (*d* = 0.31) and nonsignificant. In contrast, Stagg and Vincent (2019) reported a significant difference in the number of self-reported ASD traits between groups, with transgender and nonbinary individuals reporting more ASD traits than cisgender adults. The results were replicated by Warrier et al. (2020) in the largest study on this topic conducted to date. Using a different self-report measure (i.e., the SRS), Heylens et al. (2018) also found increased ASD traits in adults diagnosed with GD, compared to a normative sample.

## Part 2: Meta-Analyses of Studies of ASD Diagnoses and ASD Traits in GD/GI Individuals

## Method

### Sample of Studies

Our full study selection strategy is described in the method section of Part 1 and the inclusion criteria for the meta-analysis of studies of ASD diagnoses remained the same with the ones applied for the literature review (see Part 1). All the studies that reported quantitative results on the prevalence of ASD diagnoses in GD/GI people were meta-analyzed. Warrier et al. (2020) reported evidence from five datasets, all of which were independent from each other. As such, we decided to include them in the current meta-analysis as separate studies. Of the studies that reported evidence about the prevalence of ASD traits, we excluded two studies (i.e., VanderLaan, Postema, et al., 2015; Zucker et al., 2017) because they did not employ an ASD screening questionnaire. We also excluded Warrier et al.’s (2020) IMAGE and LifeLines datasets because the data reported did not allow an approximation of a standardized bias-corrected effect size to be calculated. Lastly, we excluded Leef et al.’s (2019) study because their control group was selected from a clinical population. Jones et al. (2012) and Pasterski et al. (2014) took the data of their control group from the same source (i.e., Baron-Cohen et al., 2001). To satisfy the assumption of independence of effects in the current meta-analysis, we decided to include Jones et al.’s (2012) study based on temporal criteria. Results of the meta-analysis did not change substantively when Pasterski et al.’s (2014) study was included instead (see Supplementary Material). Also, results did not change substantively when we replaced Kung’s (2020) control group from Baron-Cohen et al. (2014) with Ruzich et al's (2015), instead (see Supplementary Material).

### Meta-Analytic Procedure

To estimate the prevalence of ASD diagnoses in GD/GI people, we conducted a meta-analysis of proportions. That is a widely used method that aims to provide an accurate estimate of the frequency of a condition (Barendregt et al., 2013; Lai et al., 2019). In the current meta-analysis, the event rate of GD/GI people with a diagnosis of ASD reported in each study was transformed into a logit event rate effect size and the corresponding standard error was calculated. The transformed logit event rates were meta-analyzed using the inverse of the variance of each of the transformed rates as their study weight. The calculated pooled prevalence of ASD diagnoses in the GD/GI population and its confidence intervals were then retransformed into event rates (Lipsey & Wilson, 2001).

To conduct the meta-analysis of studies containing data on the prevalence of ASD traits in GD/GI individuals, we used Cohen’s *d* as an index of standardized mean difference in ASD traits between GD/GI and nonclinical/population-based control participants. Cohen’s *d* was calculated based on means and standard deviations provided by the authors, using Lipsey and Wilson’s (2001) web-based effect size calculator. When means and standard deviations were reported only separately for birth-assigned males at birth-assigned females within each group, combined means and standard deviations were calculated using the standard mean and standard deviation formula (Altman et al., 2000). The same formula was also used to combine the groups of transgender and nonbinary people included in Kung’s (2020) study into a single group.

To control for an overestimation of Cohen’s *d* effect size in studies with small sample size, we calculated Hedges’*g* (Hedges, 1981; Hedges & Olkin, 1985). This is the unbiased version of Cohen’s *d* and is interpreted in a similar way as Cohen’s *d* (i.e., *g* ≥ 0.20 = small effect, *g* ≥ 0.50 = moderate effect, *g* ≥ 0.80 = large effect; Cohen, 1988). Inverse variance weights were applied to effect sizes to control for sample size differences between studies (Borenstein et al., 2009; Lipsey &Wilson, 2001). 95% confidence intervals were computed as an index of variation of the estimate and the significance of the mean effect size.

The studies we selected to include in the current meta-analyses cannot be considered functionally equivalent, as they utilize a range of measures, samples, and settings. Therefore, we used a random-effects model to compute a weighted pooled estimate of the prevalence of ASD diagnoses in GD/GI individuals and a weighted mean effect size of the difference in ASD traits between GD/GI and nonclinical/population-based control participants (e.g., Borenstein et al., 2009; Lipsey & Wilson 2001; Pigott & Polanin, 2020). In the meta-analysis of studies of ASD traits, a positive effect size indicates that relative to nonclinical/population-based control people, GD/GI individuals have more ASD traits.

To quantify heterogeneity in effect sizes (or else variation in the true effect sizes) a series of measures were employed. We used the *Q* statistic to test the hypothesis that the true effect size is the same across studies. A significant *p*-value provides evidence that studies included in the meta-analysis do not share a common effect size. To examine what proportion of the observed variance reflects variation in the true effect sizes rather than sampling error, we calculated *I*^2^ values and to identify how much the true effects vary across studies we estimated prediction intervals (Borenstein et al., 2009; Borenstein et al., 2017; Higgins et al., 2003).

To examine potential sources of heterogeneity, we conducted a series of random-effects categorical analyses and meta-regression analyses. In the categorical analyses we conducted, we compared prevalence estimates of ASD diagnoses and mean differences in ASD traits between clinical-based and population-based studies. In clinical-based studies participants were recruited primarily from specialized clinics providing care to GD/GI individuals. In population-based studies, participants were recruited either randomly from the general population or from health care consortiums. Prevalence estimates of ASD diagnoses were also compared among studies that included people referred to clinics/services for gender-related issues (mainly GD), studies that included people who met diagnostic criteria or had a diagnosis of GD, GID, or GID-NOS, and studies that included GI people. Furthermore, mean differences in ASD traits were compared between studies that used primary sources of data for the control group and studies that used secondary sources. Lastly, we compared mean differences in ASD traits between studies that included primarily children (mean age < 18) and studies that included primarily adults (mean age > 18). In the meta-regression analysis we conducted, we examined the effect of age (mean) and percentage of birth-assigned males in the prevalence estimates of ASD diagnoses in GD/GI people.

If data were missing for a potential moderator, that study was excluded from the analysis. When mean age was not reported, the midpoint between the minimum and maximum of the age range was calculated (Lai et al., 2019; Loomes et al., 2017). If the mean age was reported separately for birth-assigned males at birth-assigned females, a combined mean was calculated using the standard mean and standard deviation formula (Altman et al., 2000). In the absence of a good rationale and a solid theoretical background to assume that there will be greater variation in one group compared to the other group, we used a pooled estimate of variance component to conduct our categorical analyses (e.g., Borenstein et al., 2009).

To detect the effect of publication bias, we conducted a *cumulative meta-analysis*. Studies were added one by one based on their *N* (largest to smallest), and a cumulative meta-analysis was performed with the addition of each study. An increase in the mean effect size with the addition of smaller studies provide evidence for bias (e.g., Borenstein et al., 2009). Lastly, a *one-study-removed analysis* was conducted to assess the influence of any one particular study on the results of the meta-analyses. All the analyses described above were performed using Comprehensive Meta-Analysis Software Version 3 (CMA; Borenstein et al., 2013), and prediction intervals were calculated using the spreadsheet prepared by Borenstein (2019).

## Results

### Prevalence of ASD ***Diagnoses*** in GD/GI People

**Fig. 2:**
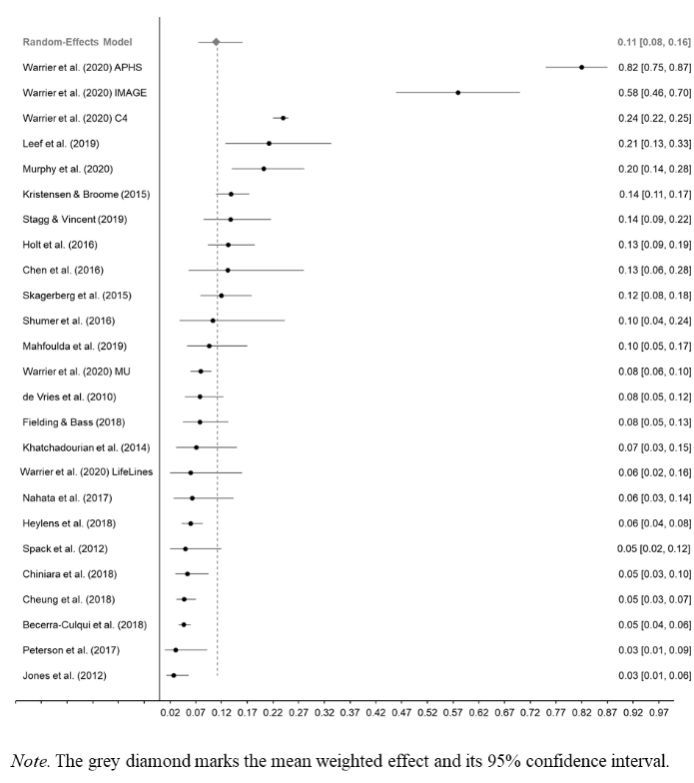
Forest Plot for Event Rate and 95% Confidence Interval for the Studies of ASD Diagnosis in GD/GI Individuals Included in the Meta-Analysis

A total of 8,662 GD/GI participants from 25 studies were included in the meta-analysis of studies of ASD diagnoses using a random-effects model (see Table S3). Of the 25 studies, 12 were conducted primarily with adults, three were conducted primarily with adolescents, nine were conducted primarily with children and adolescents, and one was conducted with children. Results from the analysis revealed that the pooled prevalence estimate of ASD diagnoses in GD/GI individuals was 0.11. The 95% confidence interval for the pooled prevalence estimate were 0.08 to 0.16 (*z* = -9.43, *p* < .001). A forest plot for this meta-analysis is depicted in Fig. 2.

The *Q*-value was 735.61, *df* = 24, *p* < .001, indicating that the effect sizes included in the analysis were significantly different from each other. The *I*^2^ statistic was 96.74, suggesting that 96.74% of the variance in the observed effects reflects variance in true effects, rather than sampling error. The variance of true effects (Tau^2^) was 1.11, the standard deviation of true effects (Tau) was 1.05, and the prediction interval was 0.01 to 0.54. Results indicate very substantial levels of heterogeneity. To investigate the sources of this heterogeneity, we conducted two random-effects categorical analyses and two univariable meta-regression analyses.

#### Moderation Analysis

As Table 1 shows, both participant type and study design were significant categorical moderators.

**Table 1:** Summary of Random-Effects Categorical Analysis Results for the Prevalence of ASD Diagnoses in GD/GI Individuals

Moderators	*Q*	*df*	*p*	Tau^2^	*k*	Prevalence	95% CI	*p*
Participants	7.80	2	0.020	0.79				
Gender-referred					9	0.09	0.05, 0.15	< 0.001
Diagnosis/Criteria					7	0.07	0.03, 0.13	< 0.001
GI					9	0.20	0.12, 0.31	< 0.001
Study design	5.99	1	0.014	0.95				
Clinical					15	0.08	0.05, 0.13	< 0.001
Population					9	0.20	0.12, 0.33	< 0.001

*Note.* Study codes are presented in Table S3. Gender-referred = participants referred to gender identity clinics/services; Diagnosis/Criteria = participants who met diagnostic criteria or had a diagnosis of GD, GID, or GID-NOS; GI = gender incongruent participants; *Q* = heterogeneity test; *df* = degrees of freedom; Tau^2^ = pooled variance component; *k* = number of studies included in each group; prevalence = event rate of ASD diagnoses; 95% CI = 95% confidence intervals.

Specifically, the prevalence estimate of ASD diagnoses was higher among GI participants than among people with a diagnosis of GD/GID/GID-NOS and people referred to specialized clinics for GD. Furthermore, the prevalence estimate from clinical-based studies was lower than that from population-based studies. However, the univariable meta-regression analyses we conducted showed that neither the mean age of participants (*Q*_*model*_ = 0.38, *df* = 1, *p* = .539; *Q*_*residual*_ = 692.73, *df* = 22, *I*^*2*^ = 96.60, *p* < .001; *R*^2^ = 0.00) nor the percentage of birth-assigned males in the sample (*Q*_*model*_ = 0.37, *df* = 1, *p* = .542; *Q*_*residual*_ = 84.44, *df* = 15, *I*^*2*^ = 81.81, *p* < .001; *R*^*2*^ = 0.00) significantly predicted the prevalence estimate of ASD diagnoses.

#### Publication Bias

To examine the impact of publication bias we conducted a cumulative meta-analysis. We found that the point estimate increased when studies with smaller sample sizes were included (see Fig. S1). Although this provides evidence that studies with small sample size introduced bias, it should be noted that even if the meta-analysis had been limited to the first 10 largest studies (*N* > 200), the pooled prevalence estimate of ASD diagnoses in GD/GI people would have been 0.08 (95% CI 0.04 to 0.13).

#### Sensitivity Analysis

Conducting a one study removed analysis, we found that none of the studies had a strong influence on the results of the meta-analysis. The pooled estimates of the prevalence of ASD diagnoses in GD/GI individuals ranged from 0.10 to 0.12 (see Fig. S2).

### Difference in ASD ***Traits*** Between GD/GI and Control Individuals

**Fig. 3:**
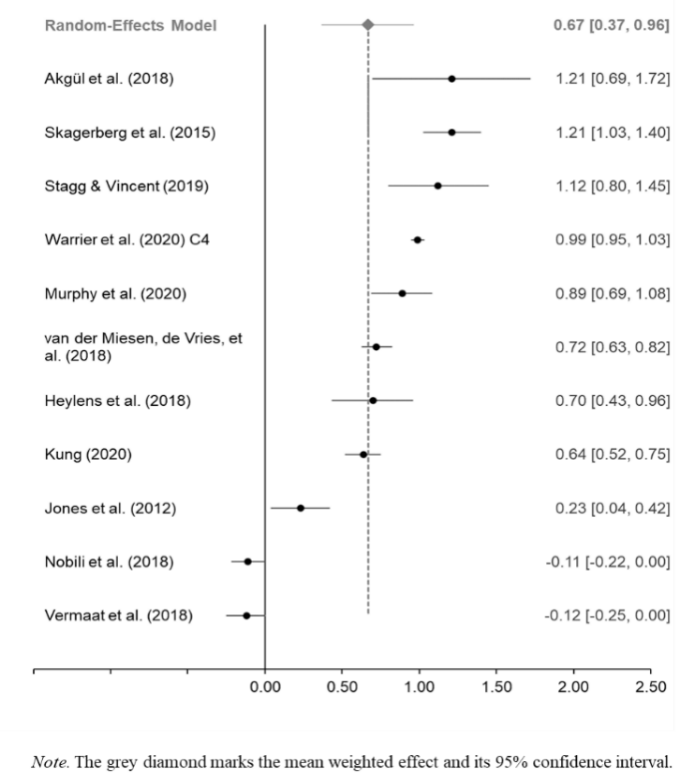
Forest Plot for Effect Sizes (g) and 95% Confidence Interval for the Studies of ASD Traits in GD/GI Individuals Included in the Meta-Analysis

A total of 4,664 GD/GI participants and 494,791 nonclinical/population-based control participants from 11 studies were included in the meta-analysis of studies of ASD traits using a random-effects model (see Table S4). Of the 11 studies, three were conducted primarily with children and adolescents and eight were conducted primarily with adults. Results from the analysis revealed that the weighted standardized mean difference in the number of reported ASD traits between GD/GI and nonclinical/population-based control participants was moderate, *g* = 0.67 (*SE* = 0.15). On average, GD/GI people reported more ASD traits than nonclinical/population-based control participants. The 95% confidence interval for the standardized mean difference was 0.37 to 0.96 (*z* = 4.38, *p* < .001). A forest plot for this meta-analysis is depicted in Fig. 3.

The *Q*-value was 608.03 (*df* = 10, *p* < .001), indicating that the true effect size is not identical in all studies. The *I*^2^ statistic was 98.36, which tells us that the 98.36% of the variance in observed effects reflects variance in true error rather than sampling error. The variance of true effects (Tau^2^) was 0.24, the standard deviation of true effects (Tau) was 0.49, and the prediction interval was − 0.50 to 1.83. Results indicate very substantial levels of heterogeneity. To investigate the sources of this heterogeneity, three random-effects categorical analyses were conducted.

#### Moderation Analysis

As Table 2 shows, none of the categorical variables we examined were significant moderators.

**Table 2:** Summary of Random-Effects Categorical Analysis Results for the Prevalence of ASD Traits in GD/GI Individuals

Moderators	*Q*	*df*	*p*	Tau^2^		*k*	*g*	*SE*	95% CI	*p*
Age group	1.71	1	0.191	0.30						
Child & adolescent						3	1.04	0.33	0.39, 1.68	0.002
Adult						8	0.54	0.20	0.15, 0.92	0.006
Control group	0.49	1	0.482	0.30						
Secondary						6	0.56	0.23	0.12, 1.01	0.013
Primary						5	0.80	0.25	0.31, 1.30	0.002
Study design	1.31	1	0.253	0.19						
Clinical						6	0.57	0.19	0.21, 0.93	0.002
Population						4	0.90	0.22	0.46, 1.34	<0.001

*Note.* Study codes are presented in Table S4. Control group = source of data; *Q* = Heterogeneity test; *df* = Degrees of freedom; *p* = Probability value; Tau^2^ = Pooled variance component; *k* = number of studies included in each group; *g* = Hedges’*g* Standardized bias-corrected effect size; *SE* = Standard error; 95% CI = 95% Confidence intervals.

Specifically, there was not a significant difference in the mean effect size between studies that used secondary and primary sources of data for their control groups. In both cases, GD/GI participants showed elevated ASD traits compared to nonclinical/population-based control participants. Likewise, no significant difference in the mean effect size was found either between clinical-based studies and population-based studies, or between studies conducted among children and studies conducted among adult cohorts.

#### Publication Bias

To examine the impact of publication bias we conducted a cumulative meta-analysis. We found that the point estimate did not increase when studies with smaller sample sizes (*N* < 200) were included (see Fig. S3). This indicates that there is no reason to assume that the inclusion of studies with smaller sample size has smaller studies has introduced bias.

#### Sensitivity Analysis

Conducting a one study removed analysis showed that none of the studies had a strong influence on the results of the meta-analysis. The overall weighted effect sizes of the difference in the number of reported ASD traits between GD/GI and nonclinical/population-based control participants ranged from 0.61 to 0.75 (see Fig. S4).

## Discussion

The review of the evidence presented in Part 1 as well as the evidence from the meta-analyses presented in Part 2 are suggestive of a link between ASD and GD/GI. The literature review, specifically, provided consistent evidence about a positive relationship between ASD traits and GD feelings in the general population (e.g., George & Stokes, 2018b; Kallitsounaki & Williams, 2020) and a high prevalence of GD/GI in autistic individuals (e.g., George & Stokes, 2018b; Hisle-Gorman et al., 2019; Pecora et al., 2020). However, we should note that despite the consistency of the findings, research in these populations is sparse. The bulk of research has focused on the prevalence of ASD/ASD traits in GD/GI people.

From the studies we reviewed it was estimated that the positive rates for ASD caseness in GD/GI people range from 1.2 to 68% (e.g., Akgül et al., 2018; Vermaat et al., 2018). Nonetheless, the variation in ASD screening questionnaires (e.g., AQ, SRS, etc.) and cutoff points used (e.g., AQ score > 26 in some studies, over 32 in other studies etc.) across different studies does not allow us to make accurate interpretations of these findings. In contrast, the evidence about the rates of autistic GD/GI people was less obscure and showed an increased prevalence of ASD diagnoses in this population (e.g., Akgül et al., 2018; Kristensen & Broome, 2015; Skagerberg et al., 2015; Warrier et al., 2020). Likewise, the results of the meta-analysis we conducted indicated that ASD frequently occurs in GD/GI individuals. Specifically, the prevalence of ASD diagnoses in this population was 11 times higher than the ASD prevalence estimate of approximately 1% in the general population (e.g., Lai et al., 2014). We should note, however, that the prediction intervals of the prevalence estimate were very wide, indicating that not all GD/GI people are affected by ASD to the same degree. Based on the characteristics of the literature pertaining to the prevalence of ASD diagnoses/ASD traits in GD/GI individuals, as discussed hereunder, wide prediction intervals were expected.

Furthermore, the findings we reviewed indicated that GD/GI *children* have higher ASD traits than control children (e.g., Akgül et al., 2018; Skagerberg et al., 2015). Yet, mixed evidence emerged about the difference in ASD traits between GD/GI *adults* and nonclinical/population-based control adults (e.g., Nobili et al., 2018; Stagg & Vincent, 2019; Warrier et al., 2020). Results of the second meta-analysis we conducted yielded a moderate (*g* = 0.67) and significant difference in the number of reported ASD traits between GD/GI and control individuals, indicating a high prevalence of ASD traits among GD/GI people. As expected, the prediction intervals were very wide denoting that not all GD/GI people report high and clinically significant levels of ASD traits.

An important point to make is that we investigated the impact of a number of potential methodological moderators on the prevalence estimate of ASD diagnoses in GD/GI people and on the mean difference in the number of ASD traits between GD/GI and control individuals. Results showed that the *study design* and *participant type* were the only significant moderators. The prevalence of ASD diagnoses was lower in clinical-based studies than in population-based studies. Also, the prevalence of ASD diagnoses was lower in people who met the diagnostic criteria or had a diagnosis of GD/GID/GID-NOS and people referred to gender clinics than in GI people. We should mention here that in clinical-based studies and studies that included people with a diagnosis or symptoms of GD information about the diagnosis of ASD was collected through medical records and in most of the studies the diagnosis was verified, whereas in studies that included GI people from the general population information was collected mainly through self-reports. This suggests that the prevalence of ASD diagnoses in GI people might be overestimated when ASD diagnosis is self-reported and that clinical-based studies might provide more precise estimates. Alternative explanations, however, are also possible (e.g., underdiagnosis of ASD in gender identity clinics/services).

To further understand and place the findings of the meta-analyses in context, a number of limitations are discussed. In both meta-analyses, the effect sizes varied substantially across studies. Although we attempted to identify factors that contribute to this variation, only two of the moderation analyses we conducted yielded significant results. We should note, however, that some of the categorical moderation analyses in this study were underpowered (Borenstein et al., 2009; Fu et al., 2011). Therefore, the absence of statistical significance does not provide strong evidence that the factors we examined do not have a contributing role in the observed variation. Furthermore, it is important to stress that the 95% prediction intervals were wide in both meta-analyses. This indicates that some of the future studies on this topic will find effect sizes that denote an overlap between ASD and GD/GI, yet other studies will not find similar results. Of course, high heterogeneity does not reduce the quality or the importance of a meta-analysis. Rather, it determines the conclusions that can be drawn from the findings and provides a better understanding of the phenomenon under investigation (Berlin, 1995; Lau et al., 1998).

The high heterogeneity observed in the current meta-analyses was not surprising, as it is common in meta-analyses that focus on ASD or GD/GI (e.g., Arcelus et al., 2015; Lai et al., 2019; Loomes et al., 2017). It likely reflects some fundamental limitations of the literature pertaining to the prevalence of ASD diagnoses/ASD traits in GD/GI individuals. First, the targeted population cannot be considered homogeneous, as it includes transgender people, nonbinary individuals, people formally diagnosed with GD, and people who do not conform to the societal expectations of their birth-assigned sex and may or may not present GD feelings. Future studies might usefully apply stricter eligibility criteria to elucidate which of the aforementioned categories are most influenced by ASD. Of equal importance is another limitation identified through the current study. That is the paucity of studies that have employed standardized diagnostic measures of ASD to identify autistic GD/GI individuals. The vast majority of information about the prevalence of ASD diagnoses in this population has been collected from patient files or it is obtained by self-reports. As such, essential information about the diagnosis itself is missing. In future studies, it might be useful to collect a copy of the diagnostic report, or when this is not possible, to collect information about the type of clinician who diagnosed ASD, the exact diagnosis received, and the age of diagnosis. Along with a formal diagnosis of ASD, standardized diagnostic tools (e.g., Autism Diagnostic Observation Schedule and Autism Diagnostic Interview-Revised; Lord et al., 2000; Rutter et al., 2003) could also be used in an attempt to elucidate the high heterogeneity we observe in the prevalence of ASD diagnoses in this population.

We should also note that although results of the second meta-analysis indicate an increased prevalence of ASD traits in GD/GI people, further research is required to examine whether *non-autistic* GD/GI people have increased ASD traits. To date, only four studies have examined this hypothesis. This was achieved either by excluding GD/GI people with a diagnosis of ASD and reanalyzing the data or by including only non-autistic GD/GI people in their samples (i.e., Akgül et al., 2018; Jones et al., 2012; Murphy et al., 2020; Warrier et al., 2020). It is also important to examine potential sex and/or gender differences in the prevalence of ASD traits in GD/GI people.

Taken together, results from the literature review and the meta-analyses indicate that the chances there is not a link between ASD and GD/GI are negligible, yet absolute conclusions about the size of the link cannot be drawn. It is well established that both autistic and GD/GI individuals are at increased risk of mental health conditions and suicidal behavior (e.g., Grant et al., 2011; Hirvikoski et al., 2016; Hofvander et al., 2009; Holt et al., 2016). When ASD and GD/GI co-occur in a person, it is possible that the risk to mental health is multiplied. Thus, it is important to understand that link. It is still unclear, for example, whether the overlap between ASD and GD/GI reflects true co-occurrence. As Williams (2017, p. 274) noted, “just because behaviorally-defined disorders A and B co-occur in a person does not mean that the underlying causes of those disorders are the same as the causes of A or B in isolation”.

One explanation for the co-occurrence of ASD with GD/GI is that some of the core features of ASD predispose an individual to develop GD feelings or disidentify with their birth-assigned gender, creating a temporal relationship between these conditions (e.g., Leef et al., 2019). For example, autistic people’s difficulty to representing mental states (namely mentalizing; e.g., Yirmiya et al., 1998) has been proposed as one of the mechanisms that could explain the link between ASD and GD/GI (Glidden et al., 2016; Jacobs et al., 2014; van der Miesen et al., 2016). Recent studies have provided some tentative, preliminary evidence in support of this hypothesis (Kallitsounaki & Williams, 2020; Kallitsounaki et al., 2021). Of course, reverse causal relationships among ASD, GD/GI, and mentalizing are certainly possible (R. J. Walsh, personal communication, 8 September, 2020) and, therefore, these links need further investigation.

Furthermore, future research might usefully investigate the development of gender identity in ASD (van Schalkwyk et al., 2015). Research on this topic is surprisingly limited and many questions remain unanswered. For example, it is unclear whether the development of gender identity in autistic and non-autistic children follows the same cognitive and developmental pathways. This is important, because it could provide a unique insight into the mechanisms that are involved in the formation and consolidation of gender identity in cisgender and GD/GI individuals. We also hope that by establishing a link between ASD and GD/GI, the current article will give new impetus to future research on the size of the link and on potential mechanisms that could explain this phenomenon. Although a number of explanations have been suggested for the co-occurrence of ASD and GD/GI (see van der Miesen et al., 2016, for a review), there is a paucity of research on them. Establishing that the link between ASD and GD/GI is “real” at the behavioral and cognitive level, will allow clinicians to start tracking the outcomes of treatment/support at a more fine-grained level (i.e., by group), rather than as a whole. Ultimately, this will promote the development of tailored services and interventions for autistic GD/GI people.

It is also important to note that the high co-occurrence between ASD and GD/GI is underrecognized among health care professionals (Murphy & Livesey, 2017). Evidence about a link between ASD and GD/GI might stimulate the development of appropriate trainings to raise their awareness (Strauss, et al., 2021), so that GD/GI people are screened for ASD and autistic people for gender related issues (Mahfouda et al., 2019; Strang, Meagher, et al., 2018). Gender related issues in autistic people could be detected by using self/parent-report measures, such as the GIDYQ-AA (Deogracias et al., 2007) and the Gender Identity Questionnaire for Children (Johnson et al., 2004) and by including a few gender related questions on a clinical intake form or a clinical interview (Strang, Meagher, et al., 2018). We should note, however, that these self-/parent-report measures have not been validated in the autistic population, so they should be used with caution. Autistic people with suspected GD should be referred to gender specialists for further assessment and support (Strang, Meagher, et al., 2018). It is essential to provide timely support and care to these people, as the burden they suffer seems to be doubly distressing (George & Stokes, 2018a; Hall et al., 2020).

## Conclusions

To our knowledge, this is the most up-to-date systematic review of the literature pertaining the overlap between ASD and GD/GI, and it is also the first meta-analysis of the prevalence of ASD diagnoses and ASD traits in GD/GI people. The findings of the current literature review and meta-analyses suggest that there is (a) a positive relationship between ASD traits and GD/GI feelings among people from the general population, (b) an increased prevalence of GD/GI in the autistic population, and (c) an increased prevalence of ASD diagnoses and ASD traits in the GD/GI population. Overall, these findings suggest the existence of a link between ASD and GD/GI that warrants the investigation of mechanisms that could explain that link and the intensification of clinical attention to autistic GD/GI individuals.

## Electronic Supplementary Material

Below is the link to the electronic supplementary material.

Supplementary Material 1

## Footnotes

1 Please note that we tend to use identity-first (i.e., autistic person) language in this article, since this is preferred by a proportion of intellectually high-functioning autistic adults. Nonetheless, there is not a universal agreement about this in the community (Kenny et al., 2016). Therefore, person-first language (i.e., person diagnosed with ASD) has been used in a few isolated places.
