# Supplementary material for: Autism Spectrum Disorder and Gender Dysphoria/Incongruence. A systematic Literature Review and Meta-Analysis
Source: J Autism Dev Disord. 2022 May 20;53(8):3103–17. doi: 10.1007/s10803-022-05517-y (PMC10313553; doi:10.1007/s10803-022-05517-y)
Supplement: Supplementary file 1 — Supplementary Material 1 [file 10803_2022_5517_MOESM1_ESM.docx]

**Supplementary Material**

1. **Prevalence of ASD *traits* in GD/GI individuals including Pasterski et al.’s (2014) study.**

When, we included Pasterski et al.’s (2014) study instead of Jones et al.’s (2012) study in the meta-analysis of studies of ASD traits in GD/GI people, results did not change substantively. Just as in the original meta-analysis, we used a random-effects model, and results revealed that the overall weighted effect size of the difference in the number of reported ASD traits between GD/GI and neurotypical/population-based participants was moderate, *g* = .66 (*SE* = 0.15, 95% CI 0.36 to 0.96, *z* = 4.28, *p* < .001). The *Q*-value was 603.94, *df* = 10, *p* < .001, indicating that the effect sizes included in the analysis were significantly different from each other. The *I*^2^ statistic was 98.34, suggesting that 98.34% of the variance in the observed effects reflects variance in true effects, rather than sampling error. The variance of true effects (Tau^2^) was 0.25, the standard deviation of true effects (Tau) was 0.50, and the prediction interval was -0.52 to 1.83.

1. **Prevalence of ASD *traits* in GD/GI individuals using Ruzich et al.’s (2015) control group in Kung’s (2020) study.**

When in Kung’s (2020) study we used the control group from Ruzich et al. (2015), instead of the control group from Baron-Cohen et al. (2014) results of the meta-analysis of studies of ASD traits in GD/GI people did not change substantively. Just as in the original meta-analysis, we used a random-effects model, and results revealed that the overall weighted effect size of the difference in the number of reported ASD traits between GD/GI and neurotypical/population-based participants was moderate, *g* = .64 (*SE* = 0.16, 95% CI 0.34 to 0.95, *z* = 4.14, *p* < .001). The *Q*-value was 643.64, *df* = 10, *p* < .001, indicating that the effect sizes included in the analysis were significantly different from each other. The *I*^2^ statistic was 98.45, suggesting that 98.45% of the variance in the observed effects reflects variance in true effects, rather than sampling error. The variance of true effects (Tau^2^) was 0.25, the standard deviation of true effects (Tau) was 0.50, and the prediction interval was -0.55 to 1.83.

**Tables**

| **Table S1** | | |
| --- | --- | --- |
| *Studies Excluded from the Literature Review* | | |
|  | Reference | Reasons for Exclusion |
| 1 | Aldridge, Z., Patel, S., Guo, B., Nixon, E., Pierre Bouman, W., Witcomb, G. L., & Arcelus, J. (2020). Long‐term effect of gender‐affirming hormone treatment on depression and anxiety symptoms in transgender people: A prospective cohort study. *Andrology.* | Quantitative study (no prevalence of ASD diagnoses/traits/caseness) |
| 2 | Baker, P. & Shweikh, E. (2016). Autistic spectrum disorders, personality disorder and offending in a transgender patient: clinical considerations, diagnostic challenges and treatment responses. *Advances in Autism*, *2*(3), 140-146. | Case report |
| 3 | Bejerot, S., Humble, M. B., & Gardner, A. (2011). Endocrine disruptors, the increase of autism spectrum disorder and its comorbidity with gender identity disorder–a hypothetical association. *International Journal of Andrology, 34*(5pt2), e350-e350. | Letter to the editor |
| 4 | Bennett, M., & Goodall, E. (2016). Towards an agenda for research for lesbian, gay, bisexual, transgendered and/or intersexed people with an Autism Spectrum Diagnosis. *Journal of Autism and Developmental Disorders*, *46*(9), 3190-3192. | Letter to the editor |
| 5 | Cain, L. K., & Velasco, J. C. (2020). Stranded at the intersection of gender, sexuality, and autism: gray’s story. *Disability & Society, 36*(3), 358-375. | Case study |
| 6 | Carlile, A. (2020). The experiences of transgender and non-binary children and young people and their parents in healthcare settings in England, UK: Interviews with members of a family support group. *International Journal of Transgender Health, 21*(1), 16-32. | Qualitative study |
| 7 | Coleman-Smith, R. S., Smith, R., Milne, E., & Thompson, A. R. (2020). ‘Conflict versus Congruence’: A Qualitative Study Exploring the Experience of Gender Dysphoria for Adults with Autism Spectrum Disorder. *Journal of Autism and Developmental Disorders, 50,* 2643–2657. | Qualitative study |
| 8 | Davidson, J., & Tamas, S. (2016). Autism and the ghost of gender. *Emotion, Space and Society, 19,* 59-65. | Review of responses to online surveys, blogs, and autobiographies |
| 9 | Di Ceglie, D. (2018). The use of metaphors in understanding atypical gender identity development and its psychosocial impact. *Journal of Child Psychotherapy, 44*(1), 5-28. | Theoretical/observational paper |
| 10 | Ehrensaft, D. (2018). Double helix rainbow kids. *Journal of Autism and Developmental Disorders*, *48*(12), 4079-4081. | Letter to the editor |
| 11 | Gallucci, G., Hackerman, F., & Schmidt, C. W. (2005). Gender identity disorder in an adult male with Asperger’s syndrome. *Sexuality and Disability*, *23*(1), 35-40. | Case report |
| 12 | George R., Stokes M. (2016) “Gender Is Not on My Agenda!”: Gender Dysphoria and Autism Spectrum Disorder. In: Mazzone L., Vitiello B. (eds) *Psychiatric Symptoms and Comorbidities in Autism Spectrum Disorder*. Springer, Cham. | Book chapter |
|  | Reference | Reasons for Exclusion |
| 13 | George, R., & Stokes, M. A. (2018). A quantitative analysis of mental health among sexual and gender minority groups in ASD. *Journal of Autism and Developmental Disorders, 48*(6), 2052-2063. | Quantitative study (no prevalence of GD/GI) |
| 14 | Glidden, D., Bouman, W. P., Jones, B. A., & Arcelus, J. (2016). Gender dysphoria and autism spectrum disorder: A systematic review of the literature. *Sexual Medicine Reviews*, *4*(1), 3-14. | Literature review |
| 15 | Hall, J. P., Batza, K., Streed, C. G., Boyd, B. A., & Kurth, N. K. (2020). Health disparities among sexual and gender minorities with autism spectrum disorder. *Journal of Autism and Developmental Disorders*, 1-7. | Mixed-methods study (no prevalence of GD/GI) |
| 16 | Hill, S. A., Thorpe, A., Petrauskaite, R., & Wilson, S. (2020). Characteristics of patients with Gender Dysphoria admitted to a secure forensic adolescent hospital*. The Journal of Forensic Psychiatry & Psychology, 31*(6), 854-867. | Quantitative study (no prevalence of ASD diagnoses/traits/caceness) |
| 17 | Hillier, A., Gallop, N., Mendes, E., Tellez, D., Buckingham, A., Nizami, A., & OToole, D. (2020). LGBTQ+ and autism spectrum disorder: Experiences and challenges. *International Journal of Transgender Health*, *21*(1), 98-110. | Qualitative study |
| 18 | Jackson-Perry, D. (2020). The autistic art of failure? Unknowing imperfect systems of sexuality and gender. *Scandinavian Journal of Disability Research, 22*(1), 221–229. | Critical analysis |
| 19 | Jacobs, L. A., Rachlin, K., Erickson-Schroth, L., & Janssen, A. (2014). Gender dysphoria and co-occurring autism spectrum disorders: Review, case examples, and treatment considerations. *LGBT Health*, *1*, 277-282. | Case study |
| 20 | Jacobs, L. A., Rachlin, K., Erickson-Schroth, L., & Janssen, A. (2016). Response to Dr. Parkinson. *LGBT Health*, *3*, 175-176. | Letter to the editor |
| 21 | James, W. H., & Grech, V. (2020). Is exposure to high levels of maternal intrauterine testosterone a causal factor common to male sex, autism, gender dysphoria, and non-right-handedness?. *Early Human Development, 141*, 104872. | Review |
| 22 | Kraemer, B., Delsignore, A., Gundelfinger, R., Schnyder, U., & Hepp, U. (2005). Comorbidity of Asperger syndrome and gender identity disorder. *European Child & Adolescent Psychiatry*, *14*(5), 292-296. | Case report |
| 23 | Kaltiala-Heino, R., Sumia, M., Työläjärvi, M., & Lindberg, N. (2015). Two years of gender identity service for minors: overrepresentation of natal girls with severe problems in adolescent development*. Child and Adolescent Psychiatry and Mental Health, 9*(1), 1-9. | Quantitative study (no prevalence of ASD diagnoses/traits/caceness) |
| 24 | Kaltiala-Heino, R., Työläjärvi, M., & Lindberg, N. (2019). Sexual experiences of clinically referred adolescents with features of gender dysphoria. *Clinical Child Psychology and Psychiatry, 24*(2), 365-378. | Quantitative study (no prevalence of ASD diagnoses/traits/caceness) |
|  | Reference | Reasons for Exclusion |
| 25 | Kuvalanka, K. A., Mahan, D. J., McGuire, J. K., & Hoffman, T. K. (2018). Perspectives of mothers of transgender and gender-nonconforming children with autism spectrum disorder. *Journal of Homosexuality*, *65*(9), 1167-1189. | Qualitative study |
| 26 | Landén, M., & Rasmussen, P. (1997). Gender identity disorder in a girl with autism-a case report. *European Child & Adolescent Psychiatry*, *6*, 170-173. | Case report |
| 27 | Lehmann, K., & Leavey, G. (2017). Individuals with gender dysphoria and autism: Barriers to good clinical practice. *Journal of Psychiatric and Mental Health Nursing, 24*(2-3), 171-177. | Essays and debates in mental health |
| 28 | Lemaire, M., Thomazeau, B., & Bonnet-Brilhault, F. (2014). Gender identity disorder and autism spectrum disorder in a 23-year-old female. *Archives of Sexual Behavior*, *43*(2), 395-398. | Case report |
| 29 | Mukaddes, N. M. (2002). Gender identity problems in autistic children. *Child: Care, Health and Development*, *28*(6), 529-532. | Case report |
| 30 | Naguy A. (2020). Autism and gender dysphoria: searching for the holy grail. *The Primary Care Companion for CNS Disorders*, *22*(2), 19l02492. | Letter to the editor |
| 31 | Nordahl-Hansen, A., Cicchetti, D. V., & Øien, R. A. (2019). A Review Update on Gender Dysphoria and ASD and Response to Corrections. *Journal of Autism and Developmental Disorders*, *49*(4), 1745-1748. | Commentary |
| 32 | Øien, R. A., Cicchetti, D. V., & Nordahl-Hansen, A. (2018). Gender dysphoria, sexuality and autism spectrum disorders: A systematic map review. *Journal of Autism and Developmental Disorders*, *48*(12), 4028-4037. | Map review |
| 33 | Parkes, G., & Hall, I. (2006). Gender dysphoria and cross-dressing in people with intellectual disability: a literature review. *Mental Retardation*, *44*(4), 260-271. | Literature review |
| 34 | Parkinson, J. (2014). Gender dysphoria in Asperger’s syndrome: A caution. *Australasian Psychiatry*, *22*(1), 84-85. | Case report |
| 35 | Parkinson, J. (2016). Gender dysphoria and autism spectrum disorders: A note of caution. *LGBT Health.* | Letter to the editor |
| 36 | Pecora, L. A., Hooley, M., Sperry, L., Mesibov, G. B., & Stokes, M. A. (2020). Sexuality and Gender Issues in Individuals with Autism Spectrum Disorder. *Child and Adolescent Psychiatric Clinics, 29*(3), 543-556. | Review |
| 37 | Perera, H., Gadambanathan, T., & Weerasiri, S. (2003). Gender identity disorder presenting in a girl with Asperger's disorder and obsessive compulsive disorder. *The Ceylon Medical Journal, 48*(2), 57–58. | Case report |
|  | Reference | Reasons for Exclusion |
| 38 | Ristori, J., Cocchetti, C., Castellini, G., Pierdominici, M., Cipriani, A., Testi, D., Gavazzi, G., Mazzoli, F., Mosconi, M., Meriggiola, M. C., Cassioli, E., Vignozzi, L., Ricca, V., Maggi, M., & Fisher, A. D. (2020). Hormonal treatment effect on sexual distress in transgender persons: 2-year follow-up data. *The Journal of Sexual Medicine, 17*(1), 142-151. | Quantitative study (no prevalence of ASD diagnoses/traits/caceness) |
| 39 | Robinow, O. (2009). Paraphilia and transgenderism: a connection with Asperger's disorder? *Sexual and Relationship Therapy*, *24*(2), 143-151. | Literature review |
| 40 | Russell, I., Pearson, B., & Masic, U. (2021). A Longitudinal Study of Features Associated with Autism Spectrum in Clinic Referred, Gender Diverse Adolescents Accessing Puberty Suppression Treatment. *Journal of Autism and Developmental Disorders, 51*(6), 2068-2076. | Longitudinal study (information about prevalence of ASD traits reported but no control group) |
| 41 | Saleem, F., & Rizvi, S. W. (2017). Transgender associations and possible etiology: A literature review. *Cureus, 9*(12), e1984. | Review |
| 42 | Selinger, D. (2018). Autism-What Does Gender Have to Do With It? *Journal of Infant, Child, and Adolescent Psychotherapy, 17*(3), 163-177. | Case report |
| 43 | Shapira, S., & Granek, L. (2019). Negotiating psychiatric cisgenderism-ableism in the transgender-autism nexus. *Feminism & Psychology, 29*(4), 494-513. | Qualitative study |
| 44 | Strang, J. F., Janssen, A., Tishelman, A., Leibowitz, S. F., Kenworthy, L., McGuire, J. K., Edwards-Leeper, L., Mazefsky, C. A., Rofey, D., Bascom, J., Caplan, R., Gomez-Lobo, V., Berg, D., Zaks, Z., Wallace, G. L., Wimms, H., Pine-Twaddell, E., Shumer, D., Register-Brown, K., Sadikova, E., Anthony, L. G., & Caplan, R. (2018). Revisiting the link: Evidence of the rates of autism in studies of gender diverse individuals. *Journal of the American Academy of Child & Adolescent Psychiatry*, *57*, 885-886. | Letter to the editor |
| 45 | Strang, J. F., Jarin, J., Call, D., Clark, B., Wallace, G. L., Anthony, L. G., Kenworthy, L., & Gomez-Lobo, V. (2018). Transgender youth fertility attitudes questionnaire: measure development in nonautistic and autistic transgender youth and their parents. *Journal of Adolescent Health, 62*(2), 128-135. | Mixed-methods study (no relevant quantitative evidence) |
| 46 | Strang, J. F., Klomp, S. E., Caplan, R., Griffin, A. D., Anthony, L. G., Harris, M. C., Graham, E. K., Knauss, M., & van der Miesen, A. I. R. (2019). Community-based participatory design for research that impacts the lives of transgender and/or gender-diverse autistic and/or neurodiverse people. *Clinical Practice in Pediatric Psychology, 7*(4), 396 – 404. | Commentary |
|  | Reference | Reasons for Exclusion |
| 47 | Strang, J. F., Knauss, M., van der Miesen, A., McGuire, J. K., Kenworthy, L., Caplan, R., Freeman, A., Sadikova, E., Zaks, Z., Pervez, N., Balleur, A., Rowlands, D. W., Sibarium, E., Willing, L., McCool, M. A., Ehrbar, R. D., Wyss, S. E., Wimms, H., Tobing, J., … Anthony, L. G. (2020). A clinical program for transgender and gender-diverse neurodiverse/autistic adolescents developed through community-based participatory design. *Journal of Clinical Child & Adolescent Psychology*, 1-16. | Mixed-methods study (no relevant quantitative evidence) |
| 48 | Strang, J. F., Meagher, H., Kenworthy, L., de Vries, A. L. C., Menvielle, E., Leibowitz, S., Janssen, A., Cohen-Kettenis, P., Shumer, D. E., Edwards-Leeper, L., Pleak, R. R., Spack, N., Karasic, D. H., Schreier, H., Balleur, A., Tishelman, A., Ehrensaft, D., Rodnan, L., E., S., Kuschner, Mandel, F., Caretto, A., Lewis, H. C., & Anthony, L. G. (2016). Initial clinical guidelines for co-occurring autism spectrum disorder and gender dysphoria or incongruence in adolescents. *Journal of Clinical Child & Adolescent Psychology, 47*(1), 105-115. | Mixed-methods study (no relevant quantitative evidence) |
| 49 | Strang, J. F., Powers, M. D., Knauss, M., Sibarium, E., Leibowitz, S. F., Kenworthy, Sadikova, E., Wyss, S., Willing, L., Caplan, R., Pervez, N., Nowak, J., Gohari, D., Gomez‑Lobo, V., Call, D., & Anthony, L. G. (2018). “They thought it was an obsession”: Trajectories and perspectives of autistic transgender and gender-diverse adolescents. *Journal of Autism and Developmental Disorders*, *48*(12), 4039-4055. | Qualitative study |
| 50 | Tateno, M., Tateno, Y., & Saito, T. (2008). Comorbid childhood gender identity disorder in a boy with Asperger syndrome. *Psychiatry and Clinical Neurosciences, 62*(2), 238. | Letter to the editor |
| 51 | Tateno, M., Teo, A. R., & Tateno, Y. (2015). Eleven-year follow up of boy with Asperger’s syndrome and comorbid gender identity disorder of childhood. *Psychiatry and Clinical Neurosciences*, *69*(10), 658-659. | Letter to the editor |
| 52 | Thrower, E., Bretherton, I., Pang, K. C., Zajac, J. D., & Cheung, A. S. (2020). Prevalence of Autism Spectrum Disorder and Attention-Deficit Hyperactivity Disorder Amongst Individuals with Gender Dysphoria: A Systematic Review. *Journal of Autism and Developmental Disorders*, *50*(3), 695-706. | Literature review |
| 53 | Turban, J. L. (2018). Potentially reversible social deficits among transgender youth. *Journal of Autism and Developmental Disorders*, *48*(12), 4007-4009. | Letter to the editor |
| 54 | Turban, J. L., & van Schalkwyk, G. I. (2018). “Gender dysphoria” and autism spectrum disorder: Is the link real? *Journal of the American Academy of Child & Adolescent Psychiatry*, 57(1), 8-9. | Critical review |
| 55 | Turban, J. L., & van Schalkwyk, G. I. (2018). Drs. Turban and van Schalkwyk reply. *Journal of the American Academy of Child & Adolescent Psychiatry, 57*(11), 887-889 | Letter to the editor |
|  | Reference | Reasons for Exclusion |
| 56 | van der Miesen, A. I. R., Cohen-Kettenis, P. T., de Vries, A. L. C. (2018). Is there a link between gender dysphoria and autism spectrum disorder? *Journal of the American Academy of Child & Adolescent Psychiatry*, 57(11), 884-885 | Letter to the editor |
| 57 | Van Der Miesen, A. I., Hurley, H., & De Vries, A. L. (2016). Gender dysphoria and autism spectrum disorder: A narrative review. *International Review of Psychiatry*, *28*(1), 70-80 | Narrative review |
| 58 | van Schalkwyk, G. I., Klingensmith, K., & Volkmar, F. R. (2015). Gender identity and autism spectrum disorders. *The Yale Journal of Biology and Medicine*, *88*(1), 81-83. | Literature review |
| 59 | Violeta, K. J., & Langer, S. J. (2017). Integration of desire, sexual orientation, and female embodiment of a transgender woman previously diagnosed with autism spectrum disorder: A case report. *Journal of Gay & Lesbian Mental Health*, *21*(4), 352-370. | Case report |
| 60 | Williams, P. G., Allard, A. M., & Sears, L. (1996). Case study: Cross-gender preoccupations in two male children with autism. *Journal of Autism and Developmental Disorders*, *26*(6), 635-642. | Case study |
| 61 | Zucker, K. J., & VanderLaan, D. P. (2018). Corrections to Øien, Cicchetti, and Nordahl-Hansen’s (2018) “Gender dysphoria, sexuality and autism spectrum disorder: A systematic map review”. *Journal of Autism and Developmental Disorders*, *48*, 4038. | Letter to the editor |

| **Table S2** | | | | | | | |
| --- | --- | --- | --- | --- | --- | --- | --- |
| *Quantitative Studies that Report Data on the Overlap Between ASD and GD/GI* | | | | | | | |
| Authors (year) | Targeted Population | Design | Focus | Age Group | Sample | Relevant Measures | Relevant Findings |
| de Vries et al. (2010) | GD/GI | Cross-sectional | ASD in GD children & adolescents | Child & adolescent | Referred to gender identity clinic (*N* = 204)  Children (*n* = 108) *M*_age_ = 8.06; *SD* = 1.82  Adolescents (*n* = 96) *M*_age_ = 13.92; *SD* = 2.29 | Diagnostic tool: DISCO-10 | 7.8% of the sample had ASD  4.7% of individuals with GID had ASD  17.0% of individuals with GID-NOS had ASD |
| Jones et al. (2012) | GD/GI | Case-control | ASD traits | Adolescent & adult | Transgender recruited online/gender identity clinic (*n* = 259)  Transgender men (*n* = 61) *M*_age_ = 34.0; range = 19-52.7  Transgender women (*n* = 198) *M*_age_ = 45.1; range = 16-75  Controls (Baron-Cohen et al., 2001; *n* = 174)  *M*_age_ = 37.0; Range = 18.1-60.0  ASD (Wheelwright et al., 2006; *n* = 125) *M*_age_ = 37.6; range = 17.6-71.1 | Screening tool: AQ-50 (cutoff scores & difference between group means)  Self-reported ASD diagnosis | 29.6% of transgender men and 5% of transgender women scored in the medium or narrow autism phenotype range  Transgender men scored significantly higher on the AQ than control women and men. The difference in the AQ score between transgender women and either control men or women was nonsignificant  2.7% of transgender individuals reported a diagnosis of Asperger syndrome or autism |
| Spack et al. (2012) | GD/GI | Retrospective chart review | Demographic & clinical data | Child, adolescent, & adult | Patients diagnosed with GID referred to a pediatric medical center (*N* = 97)  *M*_age_ = 14.8; *SD* = 3.4; range = 4-20 | ASD diagnosis: Clinic notes & self/parent report | 5.15% of patients had autism or PDD |
| Bejerot & Eriksson (2014) | ASD | Case-control | Sexuality & gender role | Adult | ASD female (*n* =24) *M*_age_ = 28.1; *SD* = 6.3  ASD male (*n* = 26) *M*_age_ = 31.8; *SD* = 7.8  Control female (*n* = 25) *M*_age_ = 27.7; *SD* = 6.7  Control male (*n* = 28) *M*_age_ = 32.9; *SD* = 7.4 | Single item measure of gender identity & androgynous behavior in childhood | Significantly more autistic individuals than non-autistic people reported an atypical gender identity  No difference was observed between autistic males and non-autistic males on being “a sissy in childhood”. Autistic females rated themselves as being more tomboyish in childhood than non-autistic females |
| Authors (year) | Targeted Population | Design | Focus | Age Group | Sample | Relevant Measures | Relevant Findings |
| Khatchadourian et al. (2014) | GD/GI | Retrospective chart review | Demographic & clinical data | Adolescent | Patients of a gender clinic diagnosed with GD (*N* = 84) *M*_age_ = 16.6, *SD* = 2.2; range = 11.4-22.5 | ASD diagnosis: Clinic notes | 7% of the sample had a diagnosis of PDD/ASD |
| Pasterski et al. (2014) | GD/GI | Case-control | ASD traits | Adult | Transgender diagnosed with GD/GID undertaking treatment at a gender clinic (*n* = 91)  MtF (*n* = 63) *M*_age_ = 45.47  FtM (*n* = 28) *M*_age_ = 27.38  Control (Baron-Cohen et al., 2001; *n* = 174) | Screening tool: AQ-50 (cutoff scores & difference between group means) | 5.5% of transgender people met the AQ threshold (> 32)  There was no significant difference in AQ score between transgender MtF and control males. Transgender FtM scored higher than control females on the AQ, but the between-group difference was nonsignificant |
| Strang et al. (2014) | ASD | Chart review | Gender variance | Child & adolescent | NT (*n* = 165) *M*_age_ = 11.87; *SD* = 3.31; range = 6-18  CBCL Normative (*n* = 1,605) *M*_age_ = 11.74; *SD* = 3.44; range = 6-18  Epilepsy/NF1 (*n* = 116) *M*_age_ = 10.12; *SD* = 2.88; range = 6-17  ADHD (*n* = 126) *M*_age_ = 9.77; *SD* = 2.95; range = 6-17  ASD (*n* = 147) *M*_age_ = 12.21; *SD* = 3.08; range = 7-18 | CBCL item 110 = Wish to be the opposite sex | The item was endorsed by parents in 0% of NT participants, 0.7 % of normative nonreferred participants, 1.7 % of participants with epilepsy or NF1, 4.8% of participants with ADHD, and 5.4% of autistic participants  Compared to the normative sample, parents of autistic participants were 7.59 times more likely to endorse the item and parents of ADHD participants were 6.64 times more likely to endorse the item |
| Kristensen & Broome (2015) | GD/GI | Cross-sectional | ASD traits | Adult | Online gender variant sample (*N* = 446)  Age range >18 | Screening tool: AQ-10 (cutoff scores)  Self-reported ASD diagnosis | 39% of the sample scored above the cutoff 6  14% of the sample reported a formal diagnosis of ASD |
| Authors (year) | Targeted Population | Design | Focus | Age Group | Sample | Relevant Measures | Relevant Findings |
| Shumer et al. (2015) | General | Cohort | Link between ASD traits & gender nonconformity | Adult | Children (*n* = 94)  ASD (*n* = 19) Year of birth, median = 1985  Controls (*n* = 75) Year of birth, median = 1985  Mothers (*n* = 198)  Fathers (*n* = 269) | SRS, 4 items from the RCGI | Higher ASD traits in children or mothers were associated with higher degrees of gender nonconformity in children |
| Skagerberg et al. (2015) | GD/GI | Cross-sectional | ASD traits | Child & adolescent | Young people with GD attending a gender identity service (*n* = 166)  *M*_age_ = 14.26; *SD* = 2.68; range = 5-18  Normative (Wigham et al., 2012; *n* = 500) | Screening tool: SRS (cutoff scores)  ASD diagnosis: Patient files | 27.1% of the GD group fell within the severe range for ASD  12.1% of the sample had a diagnosis of ASD |
| VanderLaan, Leef, et al. (2015) | GD/GI | Retrospective chart review | ASD risk factors & ASD traits | Child | Patients at the outset of assessment for GD and outpatients who had been assessed for GD (*N* = 49)  *M*_age_ = 7.19; *SD* = 2.71 | Screening tool: SRS (cutoff scores) | 44.9% of the sample fell within the clinical range for ASD |
| VanderLaan, Postema, et al. (2015) | GD/GI | Retrospective chart review | Intense/Obsessional interests | Child | Gender-referred (*n* = 534)  Siblings (n = 419) Age range = 3-12  CBCL clinic-referred (Achenbach, 1991; *n* = 1,201)  CBCL nonreferred (Achenbach, 1991; *n* = 1,201) | Screening tool (Parental ratings): CBCL items 9 (Obsessions) & 66 (Compulsions) | Gender-referred children were elevated compared to all the other groups for Item 9, and compared to siblings and nonreferred children for Item 66 |
| Chen et al. (2016) | GD/GI | Retrospective chart review | Characteristics of referrals for GD | Child & adolescent | Patients in a pediatric clinic for GD, GID, or gender identity (*N* = 38) *M*_age_ = 14.4; *SD* = 3.2 | ASD diagnosis: chart review of referrals | 13.1% of the sample had a diagnosis of ASD |
| Holt et al. (2016) | GD/GI | Cross-sectional | Demographics & associated difficulties | Child & adolescent | Referred to a gender identity development service with features of GD (*N* = 218) *M*_age_ = 14; *SD* = 3.08; range = 5-17 | ASD diagnosis: referral letters & clinical notes/reports | 13.3% of the sample had a diagnosis of ASD |
| Authors (year) | Targeted Population | Design | Focus | Age Group | Sample | Relevant Measures | Relevant Findings |
| Janssen et al. (2016) | ASD | Retrospective chart review | Gender variance | Child & adolescent | CBCL normative (Achenback et al., 2001; *n* = 1,605) *M*_age_ = 11.74; *SD* = 3.44; range = 6-18  ASD (*n* = 492) *M*_age_ = 8.96; *SD* = 2.70; range = 3-17 | CBCL item 110 = Wish to be the opposite sex | Compared to the normative sample, parents of autistic participants were 7.76 times more likely to endorse the item |
| Shumer et al. (2016) | GD/GI | Retrospective chart review | Evaluation of Asperger syndrome | Child, adolescent, & adult | Referred to a gender clinic (*N* = 39) *M*_age_ = 15.8; range = 8-20 | Screening tool: ASDS (cutoff scores)  ASD diagnosis: patient chart data | 23.1% of the sample had possibly, likely, or very likely Asperger syndrome  10.3% had a diagnosis of autism or Asperger syndrome |
| Peterson et al. (2017) | GD/GI | Retrospective chart review | Suicidality, self-harm, body dissatisfaction | Adolescent & adult | Transgender who met diagnostic criteria for GD presenting at a gender clinic (*N* = 96)  *M*_age_ = 17.1; *SD* = 2.3; range = 12-22 | ASD diagnosis: chart review | 3% of the sample had a diagnosis of ASD |
| Dewinter et al. (2017) | ASD | Case-control | Gender identity, sexual orientation, & romantic relationships | Adolescent & adult | ASD (*n* = 675) *M*_age_ = 43.2; *SD* = 13.5; range = 15-80  Controls (*n* = 8,064) *M*_age_ = 42.64; *SD* = 15.9; Range = 15-70 | Single item measure of gender identity | 15.4% of the autistic participants reported trans, nonbinary, and other/unknown gender identities. Data were not available for the control group |
| May et al. (2017) | ASD | Case-control | Gender variance | Child & adolescent | ASD (*n* = 176) *M*_age_ = 10.5; *SD* = 2.6  CBCL nonreferred (Achenbach & Rescorla, 2001; *n* = 1,605) *M*_age_ = 11.7; *SD* = 3.5; range = 6-18  CBCL clinically referred (Achenbach & Rescorla, 2001; *n* = 1,605)  *M*_age_ = 11.7; *SD* = 3.4; range = 6-18 | CBCL item 110 = Wish to be the opposite sex | Compared to parents of nonreferred children (0.7%), significantly more parents of autistic children (4%) endorsed the item. A nonsignificant difference was observed between parents of autistic children and parents of clinically referred children (4%) |
| Authors (year) | Targeted Population | Design | Focus | Age Group | Sample | Relevant Measures | Relevant Findings |
| Nahata et al. (2017) | GD/GI | Retrospective medical record review | Mental health concerns & insurance denials | Child & adolescent | Patients with ICD 9/10 codes for GD referred to a pediatric endocrinology within a gender program for hormone therapy (*N* = 79) Median _age_ = 15; range = 9-18 | ASD diagnosis: patient medical chart | 6.3% of the sample had a diagnosis of ASD. |
| Zucker et al. (2017) | GD/GI | Case control & cross validation study | Intense/obsessional interests | Child | Children referred to gender clinic service for GD (*n* = 386) *M*_age_ = 7.77; *SD* = 2.41  Clinically referred (Achenbach & Rescorla, 2001; *n* = 965)  Nonreferred (Achenbach & Rescorla, 2001; *n* = 965) | Screening tool: TRF items 9 (Obsessions) & 66 (Compulsions) | Gender-referred children were elevated compared to all the other groups for Item 9, and compared to the nonreferred children for Item 66 |
| Akgül et al. (2018) | GD/GI | Case-control | ASD traits & executive functions | Child & adolescent | Children satisfied DSM-5 criteria for GD (*n* = 25) *M*_age_ = 11.56; *SD* = 4.15  Control (*n* = 50) *M*_age_ = 11.42; *SD* = 3.91 | Screening tool: SRS (cutoff scores & difference between group means) | 68% of children with GD and 22% of control children fell within the clinical range for ASD  Children with GD had significantly more ASD traits than control children |
| Becerra-Culqui et al. (2018) | GD/GI | Retrospective & prospective cohort study | Mental health | Child & adolescent | Transgender/gender nonconforming in health care systems (*n* = 1,333)  Reference (*n* = 26,300)  Age range = 3-17 | ASD diagnosis ICD-9 Code: 299.x electronic medical records | 4.7% of the sample had a diagnosis of ASD |
| Cheung et al. (2018) | GD/GI | Retrospective audit of electronic medical records | Sociodemographic & clinical characteristics | Adolescent & adult | Transgender, nonbinary, and unassigned referred to a primary care and a secondary care gender clinic (*N* = 540) Median_age_ = 27; range = 16-72 | ASD diagnosis: electronic medical records | 4.8% of the sample had a diagnosis of ASD |
| Authors (year) | Targeted Population | Design | Focus | Age Group | Sample | Relevant Measures | Relevant Findings |
| Chiniara et al. (2018) | GD/GI | Retrospective chart review | Demographic data, clinical characteristic, and mental health | Adolescent | Adolescents presenting to a transgender clinic (*N* = 203)  AMAB (*n* = 47) *M*_age_ = 16.1; *SD* = 1.70  AFAB (*n* = 156) *M*_age_ = 16.3; *SD* = 1.63 | Self-reported ASD diagnosis: patient charts | 5.4% of the sample reported a diagnosis of ASD |
| Cooper et al. (2018) | ASD | Case-control | Gender identity & social affiliation with gender groups | Adolescent & adult | ASD female (*n* = 101) *M*_age_ = 30.38; *SD* = 12.40  ASD male (*n* = 118) *M*_age_ = 33.2; *SD* = 12.53  Control female (*n* =153) *M_age_* = 35.88; *SD* = 11.50  Control male (*n* = 114) *M*_age_ = 32.02; *SD* = 13.0 | Single item measures of gender identity & gender transition | Autistic participants were significantly more likely to be GI than control participants  Autistic participants were significantly more likely to have or be planning a gender transition than control participants |
| Fielding & Bass (2018) | GD/GI | Case note review | Pattern of referrals & characteristics | Adult | Individuals requested treatment for GD referred to clinician (*N* = 153)  Referral period 2011-2013 *M*_age_ = 34.37; *SD* = 14.95  Referral period 2014-2016 *M*_age_ = 28.70; *SD* = 13.64 | ASD diagnosis: Case notes | 7.8% of the sample had a diagnosis of ASD |
| George & Stokes (2018) | General, ASD | Case-control | Gender identity & sexual orientation | Adult | ASD (*n* = 310) *M*_age_ = 31.01; *SD* = 11.37  NT (*n* = 261) *M*_age_ = 30.20; *SD* = 11.92 | GIDYQ-AA, AQ-50, & self-reported gender identity & hormone replacement related to gender identity | ASD participants reported significantly more GD traits and a more diverse range of gender identities than NT participants. They were also more likely to use hormone replacement therapy  The rate of transgender people was 3.9% in the ASD sample and 1.9% in the NT sample  Positive association between ASD traits and GD feelings in NT adults who scored below 32 on AQ |
| Authors (year) | Targeted Population | Design | Focus | Age Group | Sample | Relevant Measures | Relevant Findings |
| Heylens et al. (2018) | GD/GI | Cross-sectional/ Retrospective chart review | Co-occurrence of ASD & GD | Adult | Patients consulted a gender clinic diagnosed with GD (*n* = 63)  AMAB (*n* = 33) *M*_age_ = 31.3; *SD* = 14.7  AFAB (*n* = 30) *M*_age_ = 22.7; *SD* = 6.5  Files of patients diagnosed with GD or GID (*n* = 532)  Normative (Constantino et al., 2012; *n* = 1,449) | Screening tools: SRS-A & AQ-50 (cutoff scores & difference between group means)  ASD diagnosis: Medical records | 27.11% of the patients scored > 60 on the SRS-A indicating mild/moderate to severe difficulties in social responsiveness  The GD group scored significantly higher on SRS-A, compared to the norm group  4.84% of GD scored above the 32 AQ cut-off point  6.02% of GD had a “certain” diagnosis of ASD |
| Nobili et al. (2018) | GD/GI | Case-control | ASD traits | Adult | Transgender from a national transgender healthcare service (*n* = 661) *M*_age_ = 28.25; *SD* = 12.25  Cisgender (*n* = 656) *M*_age_ = 28.25; *SD* = 12.25 | Screening tool: AQ-28 (cutoff scores & difference between group means) | 33.2% in the cisgender group scored ≥ 70, indicating possible ASC caseness, compared to 36.3% in the transgender group  The transgender group scored significantly lower on the AQ-28 than the cisgender group |
| van der Miesen, de Vries, et al. (2018) | GD/GI | Case-control | ASD traits | Child & adolescent | Diagnosed with GID (*n* = 490) *M*_age_ = 11.1; *SD* = 3.73  NT (Hartman et al. 2006, 2015; *n* = 2,507) *M_age_* = 10.1; *SD* = 3.73  ASD (Hartman et al. 2006, 2015; *n* = 196) *M*_age_ = 10.8; *SD* = 3.08 | Screening tool: CSBQ (cutoff scores & difference between group means) | 14.5% in the GID group had a threshold score of 38 or higher, potentially suggestive of an ASD diagnosis, compared to 3.5% in the NT sample  The GID group scored significantly higher on the CSBQ than the NT group |
| Authors (year) | Targeted Population | Design | Focus | Age Group | Sample | Relevant Measures | Relevant Findings |
| van der Miesen, Hurley, et al. (2018) | ASD | Case-control | Gender variance | Adolescent & adult | ASD adolescent (*n* = 573) *M*_age_ = 15.98; *SD* = 1.85  ASD adult (*n* = 807) *M*_age_ = 32.14; *SD* = 12.86  YSR Nonreferred adolescent (Verhulst et al., 1997; *n* = 1,016)  ASR Nonreferred adult (Achenbach, & Rescorla, 2003; *n* = 846) *M*_age_ = 29.9; *SD* = 9.5 | YSR/ASR item 110: wish to be the opposite sex | ASD adolescents were 2.12 times more likely to endorse the item, compared to nonreferred adolescents  ASD adults were 2.46 times more likely to endorse the item, compared to nonreferred adults |
| Vermaat et al. (2018) | GD/GI | Case-control | ASD symptoms | Adult | Referred to a center of expertise on GD (*n* = 326) *M*_age_ = 30.20; *SD* = 11.57  NT adults (Baron-Cohen et al., 2001; *n* = 174)  *M*_age_ = 37.0; *SD* = 7.7  NT students (Baron-Cohen et al., 2001; *n* = 840) *M*_age_ = 21.0; *SD* = 2.9  Dutch validation study NT adults (Hoekstra et al., 2008; *n* = 302) *M*_age_ = 35.68; *SD* = 6.33 | Screening tool: AQ-50 (cutoff scores & difference between group means) | 2.1% of the sample referred for GD scored above the cutoff of 32 and 9.5% above the cutoff of 26. In comparison, 2.3% of the NT adults scored above the cutoff of 32 and 8.0% above the cutoff of 26, and 2.95% of the NT students scored above the cutoff of 32 and 11.85% above the cutoff of 26. On the Dutch total AQ score, 1.2% of the GD referred group scored above the cutoff of 145  AFAB with GD scored significantly higher than NT females and NT student females on the AQ and significantly higher than NT females and NT males on the Dutch AQ total score. AMAB with GD scored significantly lower than NT males and NT student females on the AQ and significantly lower than NT males on the Dutch AQ total. All the other comparisons were nonsignificant. |
| Walsh et al. (2018) | ASD | Cross-sectional analysis | Gender identity, ASD traits, & sensory differences | Adolescent & adult | ASD (*N* = 669) *M*_age_ = 44.67; *SD* = 12.63; range = 15.92-80.14 | Single item measure of gender identity | 15% of autistic people reported trans/nonbinary identities |
| Authors (year) | Targeted Population | Design | Focus | Age Group | Sample | Relevant Measures | Relevant Findings |
| Nabbijohn et al. (2019) | General, ASD | Case-control | Gender variance | Child | NT (*n* = 2,004) *M*_age_ = 8.7; *SD* = 2.0  Clinical (*n* = 441) *M*_age_ = 9.4; *SD* = 1.9  Age range = 6-12 | GIQC/CSBQ | Positive association between characteristics of ASD and GV in the nonclinical subgroup of children  GV was associated with parent-reported clinical diagnoses of ASD, SPD, and ODD  ASD, SPD, and ODD showed significantly higher levels of parent-reported GV than nonclinical children |
| Hisle-Gorman et al. (2019) | ASD | Retrospective case-cohort | GD | Child & adolescent | ASD (*n* = 48,762)  NT (*n* = 243,810)  *M*_age_ = 8.83; *SD* = 3.44; Age range = 2-18 | Health care records ICD-9-CM codes for gender identity disorder or transsexualism | ASD children were over four times more likely to have a diagnosis indicating GD, compared to children without ASD (ASD = 0.07% vs Controls = 0.01%) |
| Leef et al. (2019) | GD/GI | Case-control | ASD traits | Child | Referred to gender identity service for GD (*n* = 61) *M*_age_ = 7.97; *SD* = 2.46; range = 4.08-12.95  Clinical comparison (*n* = 40) *M*_age_ = 9.48; *SD* = 1.81; range = 6.49-12.93 | Screening tools: SRS/SCQ (cutoff scores & difference between group means)  ASD diagnosis (PDD in DSM–IV–TR or ASD in DSM–5): Clinical file | 21.7% in the gender-referred group had a clinical range score on the SCQ, compared to 2.5% in the clinically referred group. No significant between-group differences were found in the SRS  The gender referred group scored significantly higher than the clinically referred group on the SCQ, but their scores on the SRS were similar  21.3% in the gender referred group had a diagnosis of ASD, compared to 0% in the clinically referred group |
| Mahfouda et al. (2019) | GD/GI | Retrospective chart review | Mental health correlates of ASD | Child & adolescent | Children/adolescents attending a service for gender diversity (*N* = 104) *M*_age_ = 14.62; *SD* = 1.72 | Screening tool: SRS (cutoff scores)  Self-reported ASD diagnosis | 22.1% of the sample fell in the ‘severe’ range (indicated ASD) on the DSM-5 subscale scores, and 18.3% fell in the ‘severe’ range on the SRS-2 Total scores.  9.62% of the sample reported a formal diagnosis of ASD |
| Authors (year) | Targeted Population | Design | Focus | Age Group | Sample | Relevant Measures | Relevant Findings |
| Stagg & Vincent (2019) | GD/GI | Cross-sectional | ASD traits | Adult | Online transgender/nonbinary (*n* = 109)  Transgender male *M*_age_ = 24; *SD* = 8  Transgender female *M*_age_ = 31; *SD* = 14  Nonbinary (AMAB) *M*_age_ = 29; *SD* = 14  Nonbinary (AFAB) *M*_age_ = 25; *SD* = 7  Online Cisgender (*n* = 68) Cis-male *M*_age_ = 32; *SD* = 16 Cis-female *M*_age_ = 23; *SD* = 7 | Screening tool: AQ-50 (cutoff scores & difference between group means)  Self-reported ASD diagnosis | In the non-autistic transgender and nonbinary group, 28% met the AQ cutoff score of 32, compared to 0% in the cisgender group  Both transgender and nonbinary groups scored significantly higher on AQ, compared to the cisgender group  In the transgender and nonbinary group, 14% reported an ASD diagnosis, compared to 4% in the cisgender group |
| Kallitsounaki & Williams (2020) | General | Cross-sectional | Link between ASD traits & GD | Adult | General population (*N* = 101) *M*_age_ = 36.93; *SD* = 10.11; range = 22-70 | AQ, GIDYQ-AA, & RCGI | Positive association between ASD traits, and current GD feelings and recalled childhood gender-typed behavior |
| Pecora et al. (2020) | ASD | Case-control | Gender identity, sexual orientation & adverse sexual experiences in autistic females | Adult | Autistic females (*n* = 134) *M*_age_ = 26.2; *SD* = 8.7  Non-autistic females (*n* = 161) *M*_age_ = 22.0; *SD* = 4.6 | Single item measure of gender identity | Autistic females (19.4%) were more likely to be GI than non-autistic females (8.7%) |
| Kung (2020) | GD/GI | Case-control | ASD traits, systemizing, empathizing, & theory of mind | Adult | Online transgender/nonbinary (*n* = 323) *M*_age_ = 35.83; *SD* = 15.69; range = 18-76  NT controls (Baron-Cohen et al., 2014; *n* = 3,906)  NT controls (Ruzich et al., 2015; *n* = 450,394) | Screening tool: AQ-50 (difference between group means) | Transgender men > control females  Nonbinary AFAB > control females  Transgender women = control males  Nonbinary AMAB = control males |
| Lehmann et al. (2020) | GD/GI | Cross-sectional | ASD traits | Adolescent & adult | Individuals attending or previously attended specialist gender services (*N* = 123) | Screening tools: AQ-50 & RAADS-14 (cutoff scores) | 19.5% of individuals met the AQ cutoff score of 32 and 25.4% met the RAADS-14 cutoff score of 32 |
| Authors (year) | Targeted Population | Design | Focus | Age Group | Sample | Relevant Measures | Relevant Findings |
| Murphy et al. (2020) | GD/GI | Case-control | ASD & transgender identity | Adult | Online transgender (*n* = 124) *M*_age_ = 27.31; *SD* = 10.77  Online cisgender (*n* = 603) *M*_age_ = 28.30; *SD* = 11.44 | Screening tool: AQ-50 (cutoff scores & difference between group means)  Self-reported ASD diagnosis | In the cisgender group, 10.3% met the cutoff score of 32, in comparison to 40.3% in the transgender group. Results did not change substantively when autistic participants were removed from the analysis  AQ scores were significantly higher in transgender men, compared to cisgender women and cisgender men. Transgender women showed similar AQ scores to cisgender men and cisgender women. Results did not change substantively when autistic participants were removed from the analysis  20.2% of the transgender group and 6.1% of the cisgender group reported a diagnosis of ASD |
| Nobili et al. (2020) | GD/GI | Longitudinal | Stability of ASD traits | Adult | Transgender assessed at a transgender health service (*N* = 118) *M*_age_ = 27.95; *SD* = 13.11 | Screening tool: AQ-28 (cutoff scores) | 34.7% scored above the cutoff of 70 indicating clinically significant levels of ASD traits at baseline |
| Warrier et al. (2020) | GD/GI | Case-control | Rates of ASD, other neurodevelopmental & psychiatric diagnoses, & ASD traits | Adolescent & adult | C4 age range 15-90: ASD cisgender (*n* = 27,251); NT cisgender (*n* = 484,038); ASD transgender/gender- diverse (*n* = 668); NT transgender /gender-diverse (*n* = 2,143)  MU age range 18-88: ASD cisgender (*n* = 1,031); NT cisgender (*n* = 83,950); ASD transgender/gender- diverse (*n* = 55); NT transgender/gender-diverse (*n* = 634)  IMAGE: ASD cisgender (*n* = 330); NT cisgender (*n* = 1,411); ASD transgender/gender-diverse (*n* = 36)  NT transgender/gender-diverse (*n* = 26)  APHS age range 16-90: ASD cisgender (*n* = 949); NT cisgender (*n* = 1,200); ASD transgender/gender-diverse (*n* = 133); NT transgender/gender diverse (*n* = 29)  LifeLines age > 18: ASD cisgender (*n* = 436); NT cisgender (*n* = 37,030); ASD transgender/gender diverse (*n* = 3); NT transgender/gender diverse (*n* = 50) | C4 Screening tool: AQ-10  Self-reported ASD diagnosis  MU Self-reported ASD diagnosis  IMAGE  Screening tool: AQ-50 Self-reported verified ASD diagnosis  APHS Self-reported ASD diagnosis  Life-Lines Screening tool: AQ-10  Self-reported ASD diagnosis | In the C4 dataset, transgender and gender-diverse people scored significantly higher on the AQ-10 than cisgender males and females even after accounting for the presence of ASD diagnosis  In the IMAGE dataset, transgender and gender-diverse people scored significantly higher on the AQ-50 than cisgender males and females  In the LifeLines dataset, transgender and gender-diverse people scored significantly higher on the AQ-10 than cisgender females and nominally higher than cisgender males  Across all datasets, transgender and gender-diverse individuals were 3.03 to 6.36 times more likely to be autistic than were cisgender people, after controlling for age and educational attainment |
| Kallitsounaki et al. (2021) | General | Cross-sectional | Link between ASD traits & GD | Adult | General population (*N* = 126) *M*_age_ = 20.99; *SD* = 4.10; range = 18-45 | AQ, GIDYQ-AA, & RCGI | Positive association between ASD traits, and current GD feelings and recalled childhood gender-typed behavior |
| *Note.* AFAB = assigned female at birth; AQ = Autism-spectrum Quotient; AMAB = assigned male at birth; ASC = autism spectrum conditions; ASD = autism spectrum disorder; ASDS = Asperger Syndrome  Diagnostic Scale; ASR = Adult Self-Report; CBCL = Child Behavior Checklist; CSBQ = Children’s Social Behavior Questionnaire; DISCO-10 = Diagnostic Interview for Social and Communication Disorders-  10th revision; GD = gender dysphoria; GID = gender identity disorder; GID-NOS = gender identity disorder not otherwise specified; GIDYQ-AA = Gender Identity/Gender Dysphoria Questionnaire for Adolescents and Adults; GIQC = Gender Identity Questionnaire for Children; NF1 = neurofibromatosis 1; ODD = oppositional defiant disorder; PDD = pervasive developmental disorder; RAADS-14 = The Ritvo Autism Asperger Diagnostic Scale; RCGI = Recalled Childhood Gender Identity/Gender Role Questionnaire; SCQ = Social Communication Questionnaire; SPD = Sensory processing disorder; SRS (-A) = Social Responsiveness Scale (Adult Version); NT = neurotypical; TRF = Teacher’s Report Form; YSR = Youth Self-Report. | | | | | | | |

| **Table S3** |  |  |  |  |  |  |
| --- | --- | --- | --- | --- | --- | --- |
| *Overview of Characteristics of Studies Containing Data on the Prevalence of ASD Diagnoses in GD/GI Individuals* | | | | | | |
| Author (year) | GD/GI (*N*) | Autistic people (*n*) | Mean age | Study design | % AMAB | Participants |
| de Vries et al. (2010) | 204 | 16 | 10.8 | Clinical | 56.4 | Gender-referred |
| Spack et al. (2012) | 97 | 5 | 14.8 | Clinical | 44.3 | Diagnosis/Criteria |
| Khatchadourian et al. (2014) | 84 | 6 | 16.6 | Clinical | 46.4 | Diagnosis/Criteria |
| Skagerberg et al. (2015) | 166 | 20 | 14.3 | Clinical | 37.3 | Gender-referred |
| Chen et al. (2016) | 38 | 5 | 14.4 | Clinical | 42.1 | Gender-referred |
| Holt et al. (2016) | 218 | 29 | 14.0 | Clinical | 37.2 | Gender-referred |
| Peterson et al. (2017) | 96 | 3 | 17.1 | Clinical | n.s.^c^ | Diagnosis/Criteria |
| Shumer et a. (2016) | 39 | 4 | 15.8 | Clinical | 56.4 | Gender-referred |
| Nahata et al. (2017) | 79 | 5 | 13.5^a^ | Clinical | 35.4 | Diagnosis/Criteria |
| Becerra-Culqui et al. (2018) | 1,333 | 63 | 10.0^a^ | Population | 44.1 | Diagnosis/Criteria |
| Chiniara et al. (2018) | 203 | 11 | 16.3^b^ | Clinical | 23.2 | Gender-referred |
| Leef et al. (2019) | 61 | 13 | 8.0 | Clinical | 73.8 | Diagnosis/Criteria |
| Mahfouda et al. (2019) | 104 | 10 | 14.6 | Clinical | 24.0 | Gender-referred |
| Jones et al. (2012) | 259 | 7 | 42.5^b^ | Clinical & Population | 76.4 | GI |
| Kristensen & Broome (2015) | 446 | 62 | 46.5^a^ | Population | n.s.^c^ | GI |
| Cheung et al. (2018) | 540 | 26 | 44.0^a^ | Clinical | n.s.^c^ | Gender-referred |
| Fielding & Bass (2018) | 153 | 12 | 30.7^b^ | Clinical | 63.4 | Gender-referred |
| Heylens et al. (2018) | 532 | 32 | n.s. | Clinical | 66.0 | Diagnosis/Criteria |
| Stagg & Vincent (2019) | 109 | 15 | 26.5^b^ | Population | 34.9 | GI |
| Murphy et al. (2020) | 124 | 25 | 27.3 | Population | 38.7 | GI |
| Warrier et al. (2020) C4 | 2,811 | 668 | 25.4 | Population | n.s.^c^ | GI |
| Author (year) | GD/GI (*N*) | Autistic people (*n*) | Mean age | Study design | % AMAB | Participants |
| Warrier et al. (2020) MU | 689 | 55 | 22.4 | Population | n.s.^c^ | GI |
| Warrier et al. (2020) IMAGE | 62 | 36 | 29.7 | Population | n.s.^c^ | GI |
| Warrier et al. (2020) APHS | 162 | 133 | 35.2 | Population | n.s.^c^ | GI |
| Warrier et al. (2020) LifeLines | 53 | 3 | 47.9 | Population | n.s.^c^ | GI |
| *Note.* All studies included in this table were meta-analyzed; Diagnosis/Criteria = people who have an official diagnosis or meet GD/GID/GID-NOS criteria; GD = gender dysphoria; GI = gender incongruent; GID = gender identity disorder; GID-NOS = gender identity disorder not otherwise specified; Gender-referred = people referred to clinics/services for gender related issues (mainly GD); AMAB = assigned male at birth; n.s. = not specified.  ^a^ Mean age was calculated by taking a midpoint between the minimum and maximum of the age range. ^b^ Combined mean age was calculated from the data provided by the authors. ^c^ Information about birth-assigned sex was not reported for the total number of participants who took part in the study. | | | | | | |

| **Table S4** | | | | | | |
| --- | --- | --- | --- | --- | --- | --- |
| *Overview of Characteristics of Studies Containing Data on the Prevalence of ASD Traits in GD/GI Individuals* | | | | | | |
| Author (year) | Study design | Number of participants per group | Age group | Control group | Mean scores (*SD*) | Direction of effect (Hedges’*g*) |
| Skagerberg et al. (2015) | Clinical | *n_case_* = 166  *n_control_* = 500 | Child & adolescent | Secondary | *M*_case_ = 58.51 (37.58)^a^  *M*_control_ = 29.8 (16.7) | Cases > Controls (1.21) |
| Akgül et al. (2018) | Clinical | *n_case_* = 25  *n_control_* = 50 | Child & adolescent | Primary | *M*_case_ = 70.36 (16.72)  *M*_control_ = 49.78 (16.95) | Cases > Controls (1.22) |
| van der Miesen, de Vries, et al. (2018) | Clinical | *n_case_* = 490  *n_control_* = 2,507 | Child & adolescent | Secondary | *M*_case_ = 20.58 (15.71)  *M*_control_ = 11.69 (11.49) | Cases > Controls (0.72) |
| Jones et al. (2012) | Clinical & population | *n*_case_ = 259  *n_control_* = 174 | Adult | Secondary | *M*_case_ = 18.15 (7.97)^a^  *M*_control_ = 16.4 (6.3) | Cases > Control (0.23) |
| Heylens et al. (2018) | Clinical | *n_case_* = 58  *n*_control =_ 1,449 | Adult | Secondary | *M*_case_ = 52.53 (22.48)  *M*_control_ = 36.74 (22.66) | Cases > Controls (0.70) |
| Nobili et al. (2018) | Clinical | *n_case_* = 656  *n_control_* = 656 | Adult | Primary | *M*_case_ = 65.77 (11.81)  *M*_control_ = 66.88 (8.48) | Cases < Controls (-0.11) |
| Stagg & Vincent (2019) | Population | *n_case_* = 109  *n_control_* = 68 | Adult | Primary | *M*_case_ = 28.72 (10.06)^a^  *M*_control_ = 18.41 (7.39)^a^ | Cases > Controls (1.12) |
| Vermaat et al. (2018) | Clinical | *n_case_* = 326  *n*_control_ = 840 | Adult | Secondary | *M*_case_ = 16.79 (6.96)  *M*_control_ = 17.6 (6.4) | Cases < Controls (-0.12)^b^ |
| Murphy et al. (2020) | Population | *n_case_* = 124  *n*_control_ = 603 | Adult | Primary | *M*_case_ = 28.04 (11.37)^a^  *M*_control_ = 19.70 (8.96)^a^ | Cases > Controls (0.89) |
| Kung (2020) | Population | *n_case_* = 308  *n*_control_ = 3,906 | Adult | Secondary | *M*_case_ = 23.20 (8.50)^a^  *M*_control_ = 18.20 (7.82)^a^ | Cases > Controls (0.64) |
| Warrier et al. (2020) C4^c^ | Population | *n_case_* = 2,143  *n*_control_ = 484,038 | Adult | Primary | *M*_case_ = 5.56 (2.69)  *M*_control_ = 3.32 (2.26)^a^ | Cases > Controls (0.99) |
| *Note.* All studies included in the table were meta-analyzed.  ^a^ Means and *SD*s were calculated from the data reported by the authors. ^b^ Vermaat et al. (2019) reported that the difference between the sample referred for GD and the comparison group of NT students in AQ score was *d* = -0.28. When we calculated this effect from the means and *SD*s provided by the authors in the original publication, we found that the *d* was -0.12. ^c^ Means and *SD*s refer to non-autistic participants. | | | | | | |

**Figure S1**

*Cumulative Forest Plot of Studies Included in ASD Diagnosis Meta-Analysis*

**
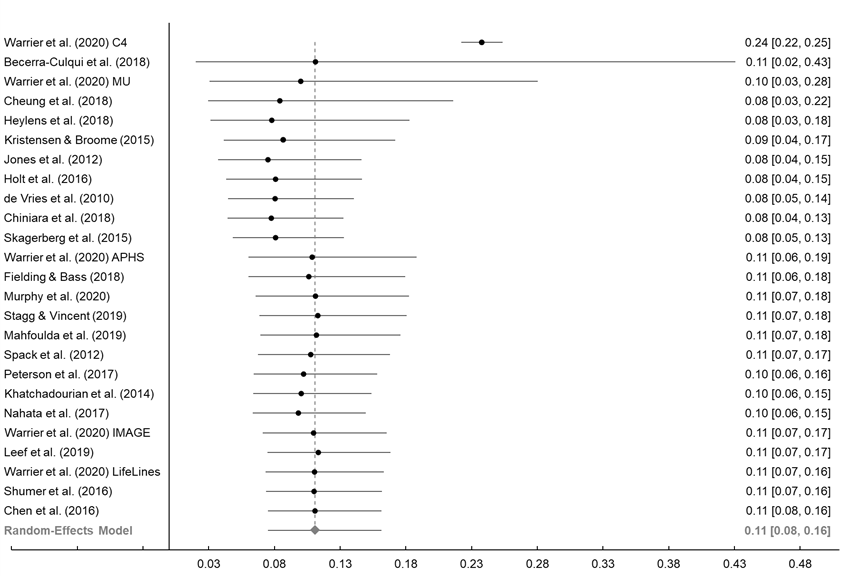
**

*Note.* Each row shows the pooled prevalence estimate of ASD diagnoses in GD/GI people and its 95% confidence interval when that row’s study is included in the meta-analysis. The grey diamond marks the pooled prevalence estimate and its 95% confidence interval when all studies are included in the meta-analysis.

**Figure S2**

*Forest Plot of Pooled Estimate Prevalence Estimates of ASD Diagnoses After One Study Removed*
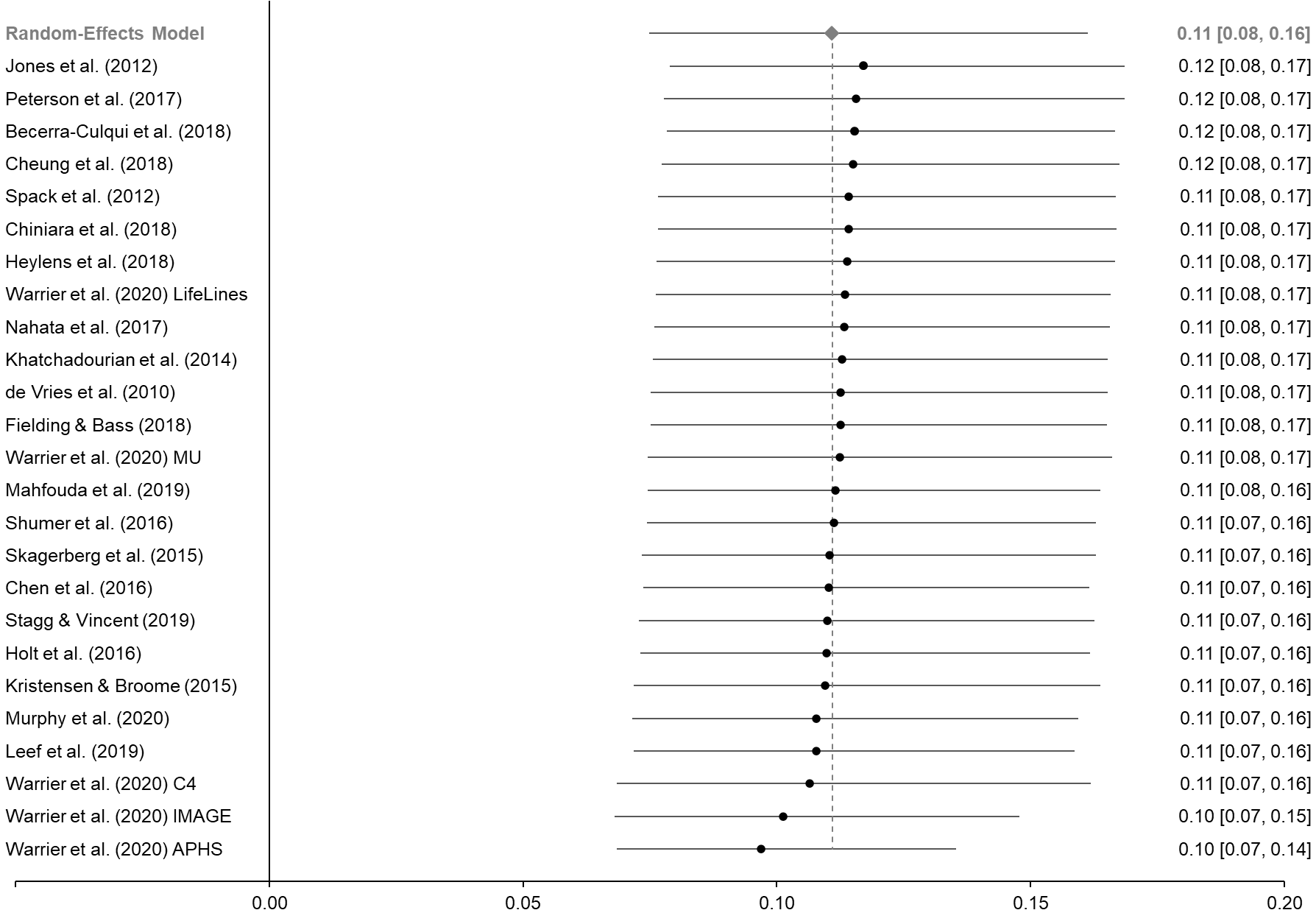


*Note.* Each row shows the pooled prevalence estimate of ASD diagnoses in GD/GI people and its 95% confidence interval when that row’s study was removed from the meta-analysis. The grey diamond marks the mean weighted effect and its 95% confidence interval when all studies were included in the meta-analysis.

**Figure S3**

*Cumulative Forest Plot of Studies Included in ASD Traits Meta-Analysis
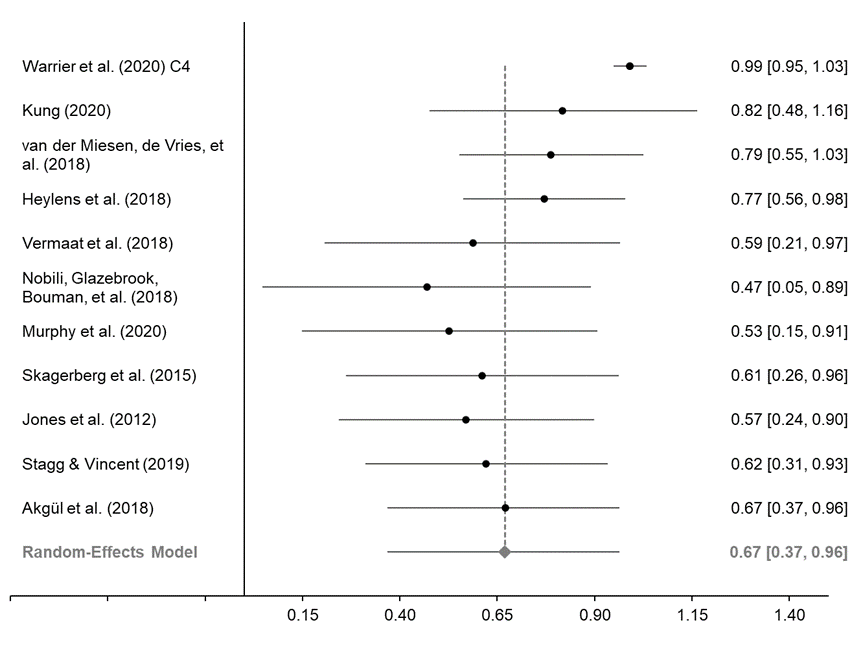
*

*Note.* Each row shows the overall weighted effect size of the difference in the number of reported ASD traits between GD/GI and neurotypical/population-based participants and its 95% confidence interval when that row’s study is included in the meta-analysis. The grey diamond marks the pooled prevalence estimate and its 95% confidence interval when all studies are included in the meta-analysis.

**Figure S4**

*Forest Plot of Hedges’ g After One Study Removed*


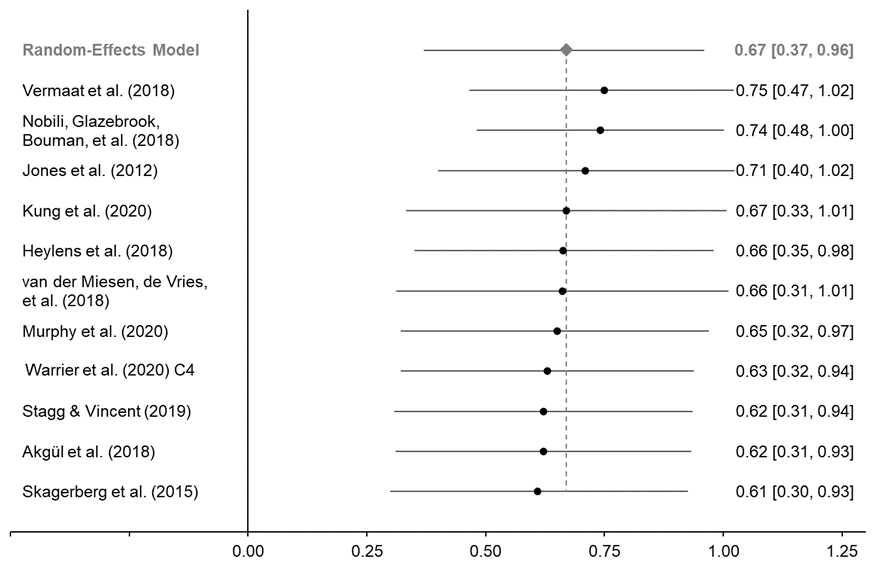


*Note.* Each row shows the overall weighted effect size of the difference in the number of reported ASD traits between GD/GI and neurotypical/population-based participants and its 95% confidence interval when that row’s study was removed from the meta-analysis. All *p* values were ≤ .003. The grey diamond marks the mean weighted effect and its 95% confidence interval when all studies were included in the meta-analysis.
